# EFFECTOR OF TRANSCRIPTION factors are novel plant‐specific regulators associated with genomic DNA methylation in Arabidopsis

**DOI:** 10.1111/nph.15439

**Published:** 2018-09-25

**Authors:** Francesca Tedeschi, Paride Rizzo, Bui Thi Mai Huong, Andreas Czihal, Twan Rutten, Lothar Altschmied, Sarah Scharfenberg, Ivo Grosse, Claude Becker, Detlef Weigel, Helmut Bäumlein, Markus Kuhlmann

**Affiliations:** ^1^ Leibniz Institute of Plant Genetics and Crop Plant Research (IPK) 06466 Seeland OT Gatersleben Germany; ^2^ Department of Bioinformatics Martin‐Luther‐University 06120 Halle Germany; ^3^ Department of Molecular Biology Max Planck Institute for Developmental Biology 72076 Tübingen Germany; ^4^ Gregor Mendel Institute of Molecular Plant Biology 1030 Vienna Austria

**Keywords:** *Arabidopsis thaliana*, DNA methylation, DNA repair, EFFECTOR OF TRANSCRIPTION (ET), methylome

## Abstract

- Plant‐specific EFFECTORS OF TRANSCRIPTION (ET) are characterised by a variable number of highly conserved ET repeats, which are involved in zinc and DNA binding. In addition, ETs share a GIY‐YIG domain, involved in DNA nicking activity. It was hypothesised that ETs might act as epigenetic regulators.
- Here, methylome, transcriptome and phenotypic analyses were performed to investigate the role of ET factors and their involvement in DNA methylation in *Arabidopsis thaliana*.
- Comparative DNA methylation and transcriptome analyses in flowers and seedlings of *et* mutants revealed ET‐specific differentially expressed genes and mostly independently characteristic, ET‐specific differentially methylated regions. Loss of ET function results in pleiotropic developmental defects.
- The accumulation of cyclobutane pyrimidine dimers after ultraviolet stress in *et* mutants suggests an ET function in DNA repair.

## Introduction

Plant development depends on complex regulatory interactions, including the orchestrated coordination of numerous transcriptional networks. While interactions of transcription factors with DNA are essential for regulating gene expression, these are often modified through epigenetic mechanisms such as DNA methylation and histone modifications (Du *et al*., 2015).

Previous work on plant embryogenesis has led to the isolation of a plant‐specific class of gene regulators (Raventos *et al*., 1998; Ellerstrom *et al*., 2005; Ivanov *et al*., 2008) with the founding members known as EFFECTOR OF TRANSCRIPTION (ET). Overexpression studies have shown that ET factors can affect diverse developmental processes, such as seed germination and xylem differentiation (Ellerstrom *et al*., 2005; Ivanov *et al*., 2008). ET proteins share highly conserved cysteine–histidine domains with zinc‐ and DNA‐binding repeats. These repeats are also found in nonflowering plants such as the moss *Physcomitrella patens*, demonstrating their evolutionary conservation. ET1 and ET2‐GFP fusion proteins are detectable in the nucleus (Ivanov *et al*., 2008). In addition to their functional DNA‐binding ET repeats (Ellerstrom *et al*., 2005), ET factors share a characteristic DNA single‐strand nuclease domain (GIY‐YIG) with structural similarity to that of bacterial UVRC proteins (Dunin‐Horkawicz *et al*., 2006) and homing nucleases (Stoddard, 2005; Liu *et al*., 2013). The bacterial UVRC protein is essential for DNA excision repair (Moolenaar *et al*., 1998a,b). It is targeted to ultraviolet (UV)‐induced DNA lesions such as thymidine‐dimers, and introduces two single‐strand cuts eight bases upstream and four bases downstream of a lesion. The two single‐strand cuts are made by two structurally distinct domains: a C‐terminal domain consisting of an Endonuclease V (EndoV) and a Helix‐hairpin‐Helix (HhH) domain, which are required for the 5′‐cut, and an N‐terminal GIY‐YIG domain, which inserts the 3′‐nick (Van Roey *et al*., 2002). The sequence similarity between plant ET factors and UVRC is restricted to this single‐strand cutting GIY‐YIG domain, suggesting that an ancestral bacterial GIY‐YIG domain has been recruited by ET proteins and combined with the DNA‐binding ET repeats to create a novel plant‐specific regulatory protein (Ivanov *et al*., 2008). The single‐strand cleavage function of the Arabidopsis ET2 GIY‐YIG domain has been confirmed by substitution and complementation of the corresponding domain of the *Escherichia coli* UVRC protein (Ivanov *et al*., 2008). On the transcriptional level, a sevenfold upregulation of ET2 was described in response to ionising radiation in Arabidopsis plants (Culligan *et al*., 2006). This upregulation was not detectable in plants deficient for ATAXIA‐TELANGIECTASIA MUTATED (ATM), a sensor for DNA damage. This observation was driving our hypothesis that ET factors are involved in DNA repair.

The HhH domain, the second DNA‐nicking domain in the UVRC protein, which is structurally distinct from the GIY‐YIG domain, has been considered as the ancestral protein domain for two related epigenetic plant regulators, the DNA glycosylase DEMETER (DME) (Choi *et al*., 2002) and the REPRESSOR OF SILENCING 1 (ROS1) (Gong *et al*., 2002; Morales‐Ruiz *et al*., 2006). DME can introduce single‐strand nicks as part of a DNA demethylation pathway, whereas ROS1 represses homology‐dependent transcriptional silencing by demethylation of the target promoter (Gong *et al*., 2002). The importance of DNA methylation as an epigenetic marker required for several developmental phases such as seed development and germination was described recently (Kawakatsu *et al*., 2017). Several regions have been identified in which dynamic control of DNA methylation and transcriptional reactivation is contributing to reproductive development (Borges *et al*., 2012). Although basic molecular analysis of ETs has been performed, the functional context *in planta* remains elusive.

Considering the structural and functional similarities between DME/ROS1 and the ET factors, we hypothesised that ETs are involved in regulation of DNA methylation based on their single‐strand cleavage function. Here, we performed whole‐genome DNA methylation analyses in flowers of *et1* and *et2* mutants to gain insights into the function of these proteins. From combining methylome data with transcriptional profiles and with extensive phenotypic analyses in different organs and tissues, we propose that Arabidopsis ET factors constitute a new class of epigenetic regulators involved in stable inheritance of DNA methylation patterns.

## Materials and Methods

### Plant material, mutant characterisation and complementation

*Arabidopsis thaliana* (L.) Heynh. plants of accession Columbia‐0 (Col‐0) for *et1‐1*,* et2‐3*,* et1‐1 et2‐3* and Wassilewskija‐2 (Ws‐2) accession for *et2‐1* were grown in growth chambers under a 16‐h photoperiod at 22°C and 60% humidity. Alternatively, seeds from each line were plated on Murashige and Skoog (MS; Murashige & Skoog, 1962) agar plates, supplemented with the appropriate antibiotic or herbicide when required. Seedlings were grown in growth chambers (Percival Scientific, Perry, IA, USA), under a 16‐h photoperiod at 22°C. Green seedlings were transferred to soil and grown under the same conditions as for the mature plants.

**Figure 1:**
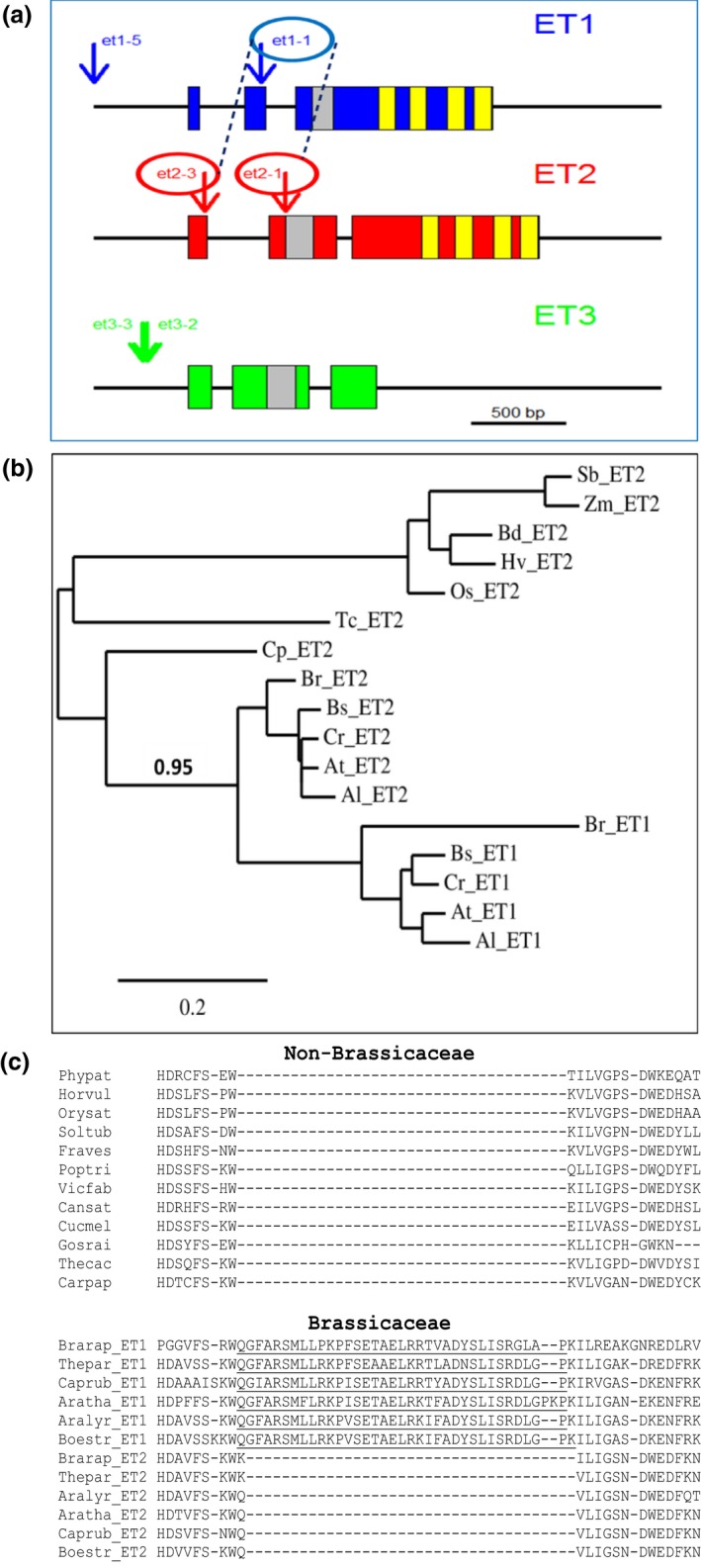
EFFECTORS OF TRANSCRIPTION (ET) gene family features. (a) Gene models and positions of T‐DNA insertions of the *ET* gene family in *Arabidopsis thaliana*. The positions of T‐DNA insertions are indicated by arrows. ET repeats and the GIY‐YIG single‐strand cutting domain are given in yellow and grey, respectively. The dashed lines indicate the alleles which have been combined as homozygous double mutants. (b) Phylogenetic tree of ET proteins and ET1‐specific second exon in Brassicaceae. Protein sequences were identified via Blastp in the Phytozome databases (phytozome.jgi.doe.gov) for *Arabidopsis lyrata* v1 (Al), *A. thaliana* TAIR9 (At), *Boechera stricta* v1 (Bs), *Brachypodium distachyon* v2 (Bd), *Brassica rapa* v1 (Br), *Capsella rubella* v1 (Cr), *Carica papaya* r.Dec2008 (Cp), *Oryza sativa* v7 (Os), *Sorghum bicolor* v2 (Sb), *Theobroma cacao* CGDv1 (Tc) and *Zea mays* AGPv3 (Zm) and classified after sequence alignment into ET1 and ET2 types. The HRT gene (*Hordeum vulgare*; GenBank accession CAA04677), which is an ET2 type gene, was added. ET1 type genes could only be identified in Brassicaceae genomes (Al, At, Bs, Br, Cr), which evolved after the α‐whole genome duplication event *c*. 47 million yr ago (Hohmann *et al*., 2015), while all genomes encode ET2 types. A phylogenetic tree was calculated using the web service at www.phylogeny.fr (‘one click’ method with Gblocks for curation of the muscle alignment; Dereeper *et al*., 2008). The phylogenetic tree clearly shows that ET1 types and ET2 types are sister groups within the Brassicaceae. The bootstrap value is given for the node separating Brassicaceae from other plant species and for splitting of ET1 and ET2 types of the Brassicaceae species. (b) Scale bar indicates the proportion of amino acids changing along each branch per amino acid used for the alignment. (c) Amino acid sequence alignment using the muscle program of various Brassicaeceae and non‐Brassicaceae species. Phypat, *Physcomitrella patens*; Horvul, *H. vulgare*; Orysat, *O. sativa*; Soltub, *Solanum tuberosum*; Fraves, *Fragaria vesca*; Poptri, *Populus trichocarpa*; Vicfab, *Vicia faba*; Cansat, *Cannabis sativa*; Cucmel, *Cucumber melon*; Gosrai, *Gossypium raimondii*; Thecac, *T. cacao*; Carpap, *C. papaya*; Brarap, *B. rapa*; Thepar, *Thellungiella parvula*; Caprub, *C. rubella*; Aratha, *A. thaliana*; Aralyr, *A. lyrata*; Boestr, *B. stricta*.

T‐DNA insertion lines *et1‐1 and et2‐3* generated in the genetic background of Col‐0 were obtained from the SALK T‐DNA collection (Alonso *et al*., 2003) and designated as *et1‐1* (SALK_000422) and *et2‐3* (SALK_151861). The Ws‐2‐derived *et2‐1* mutant (Ivanov *et al*., 2008, 2012) was isolated from the collection of the *Arabidopsis* Knock‐out Facility (Sussman *et al*., 2000) at the University of Wisconsin Biotechnology Center, following a pool screening for insertion in the *ET2* gene in the Ws‐2 background (Krysan *et al*., 1999) and has been back crossed six‐times to the Col‐0 ecotype. The positions of the T‐DNA insertions are summarized in Fig. 1(a). Homozygous single mutants, *et1‐1, et2‐1* and *et2‐3*, have been isolated and homozygous double mutants, *et1‐1 et2‐1* and *et1‐1 et2‐3*, have been generated. Absence of full‐length mRNA was confirmed by qualitative PCR spanning the T‐DNA insertion site. Double mutants were generated by crossing the mutant *et1‐1* with the *et2‐1* and *et2‐3* mutants, respectively. Primer sequences are provided in Supporting Information Table S1. To complement the *et1‐1* mutation, a Col‐0‐derived *ET1* genomic fragment including sequences 1228 bp upstream of the start codon and 792 bp downstream of the stop codon was PCR amplified using Platinum Taq High Fidelity (Invitrogen) and resequenced. The gene fragment was cloned into the pDONR/Zeo vector using the BP reaction (Gateway^®^ BP Clonase^®^ Enzyme Mix, Invitrogen) and transferred into the pBGW destination vector (Karimi *et al*., 2007) using the LR reaction (Gateway^®^ LR Clonase^®^ Enzyme Mix, Invitrogen). The complementation construct was introduced into *Agrobacterium tumefaciens* strain GV2260 by freeze–thaw transformation (Chen *et al*., 1994). The floral dip method (Clough & Bent, 1998) was used for plant transformation.

UV stress was applied using 1‐wk‐old seedlings and placing them for 15 min at 30 cm from an MBR UV‐C mobile room sterilizer, mediating 165 µW cm^−2^ m^−1^ UV‐C light (253.7 nm).

### RNA extraction, cDNA synthesis

Total RNA was isolated from 10‐d‐old seedlings grown under sterile conditions on solid MS medium and from flower buds at the 12c–14 stage (Smyth *et al*., 1990).Total RNA was isolated from 100 mg of plant material using the RNeasy Plant Mini kit as described in the manufacturer's protocol (Qiagen) dissolved in 30 μl DEPC‐treated water and treated with DNaseI (Roche). Total RNA concentration was quantified using a Nanodrop^®^ ND‐1000 spectrophotometer (NanoDrop Technologies Inc., Waltham, MA, USA) and the quality and integrity was assessed by running 1 μl of every sample on an Agilent 2100 Bioanalyzer (Agilent Technology Inc., Waldbronn, Germany). First‐strand cDNA was synthesized by reverse transcription from total RNA using the RevertAid H Minus First strand cDNA synthesis kit (Fermentas, Vilnius, Lithuania).

### Quantitative real‐time PCR

Quantitative real‐time measurements were performed using SYBR Green Master Mix reagent in an ABI Prism 7700 Sequence Detection System (Applied Biosystems), according to the manufacturer's instructions. For each condition, three technical replicates and three biological replicates were used. Transcript levels were determined by quantitative real‐time PCR (RT‐PCR) and the raw threshold cycle values (*C*_T_) for all samples were normalised against *C*_T_ values obtained for the reference transcript of the *ACTIN11* gene using qbase software (Biogazelle, Ghent, Belgium). Primers used in this work were designed with the QuantPrime tool (Arvidsson *et al*., 2008) and are listed in Table S1.

### RNA deep sequencing

Strand‐specific cDNA libraries for Illumina Next Generation Sequencing were generated from triplicate biological samples (10‐d‐old seedlings and flower buds at the 12c–14 stage). For detailed description see Methods S1.

### DNA methylation analysis

For detailed description of DNA methylation analysis see Methods S1.

### Immunodetection of cyclobutane pyrimidine dimers (CPDs)

After 3 h of regeneration samples were taken from the aerial tissue and genomic DNA was extracted using a DNeasy Plant Mini kit as described in the manufacturer's protocol (Qiagen). In total, 1 μg of DNA was spotted on an Amersham Hybond N^+^ Nylon membrane (GE Healthcare, Little Chalfont, UK). Immunodetection was performed according to the manufacturer's description in TBST/5% milk powder using the Anti‐Thymine Dimer primary antibody (H3) from Abcam (ab10347, Cambridge, UK) and ECL anti‐mouse IgG horseradish peroxidase‐linked whole secondary antibody (NA931V, GE Healthcare). Signals were detected using Clarity Western ECL substrate (Bio‐Rad) and Amersham Hyperfilm ECL (GE Healthcare). Quantification of signals was performed from three independent experiments using the quantity one 4.5.2 software (Bio‐Rad). For quantification the adjusted volume intensity × mm^2^/unstressed Col‐0 background signal was calculated.

### Microscopy techniques

For detailed description of microscopy techniques see Methods S1.

## Results

### Gene family evolution

The *ET* gene family in *A. thaliana* (Fig. 1a) comprises three members, *ET1* (AT4G26170), *ET2* (AT5G56780) and *ET3* (AT5G56770). *ET1* and *ET2* encode all characteristic ET sequence motifs, including the typical cysteine‐rich ET repeats and the GIY‐YIG domain, whereas *ET3* is a partial tandem duplication of *ET2* lacking the C‐terminal ET repeats. This 3′ truncated gene is located downstream of *ET2* and considered a nonfunctional pseudogene. Here, we focused our analysis on *ET1* and *ET2*, which contain the name‐giving ET‐domain. *ET* genes are exclusively found in plants, suggesting their involvement in plant‐specific processes. A phylogenetic tree identified the *ET2*‐type gene as ancestral, dating back to the common ancestor of mosses and seed plants (Fig. 1b). *ET2* consists of three exons of which the second encodes the GIY‐YIG domain and the third contains the characteristic ET repeats. *ET1* probably resulted from deletion of the second intron of *ET2* and an insertion of a complete exon into the first intron of *ET2* (Fig. 1c). Acquisition of the second exon, characteristic for *ET1* genes, is only found in species of the family Brassicaceae (Fig. 1c). The evolutionary origin of *ET1* might be the α‐whole genome duplication event in this family (Hohmann *et al*., 2015).

### Whole genome analysis of differentially methylated regions in *et* mutants

**Figure 2:**
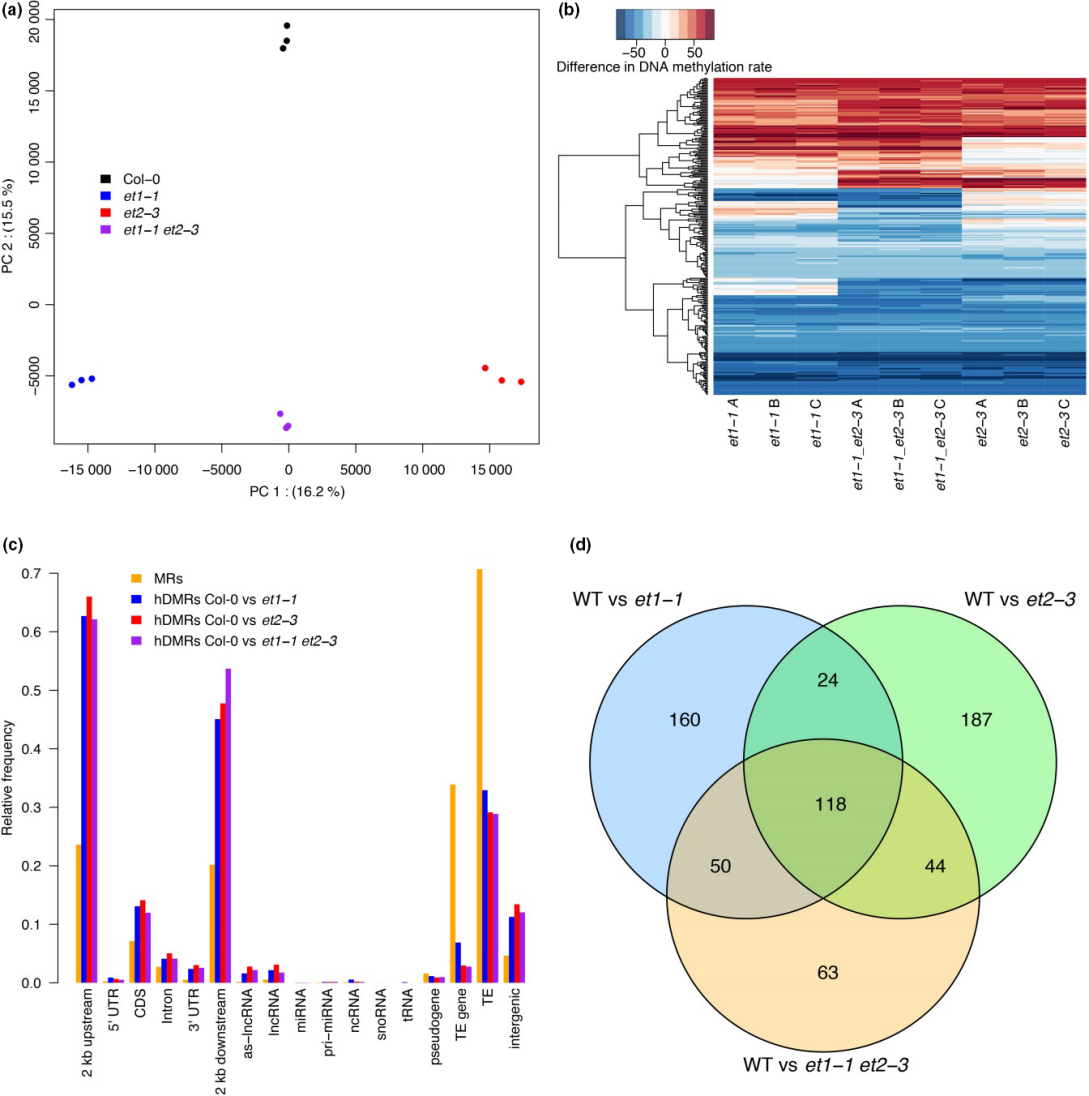
Genome‐wide methylation analysis of *Arabidopsis thaliana et* mutants in flower. (a) Principal component (PC) analysis of methylation rates within highly differentially methylated regions (hDMRs). For each hDMR, the average methylation rate was calculated per sample from the methylation rates of all cytosines contained within the region. Percentages indicate the amount of variance explained by the respective PC. (b) Gains and losses of methylation in *et1‐1*,* et1‐1 et2‐3* and *et2‐3* hDMRs of all contexts (CG, CHG, CHH) . Each line in the heat map represents an hDMR. Gains and losses are expressed as difference of the methylation rate in the mutant to the average of the three Col‐0 replicates. (c) Annotation of cytosines in methylated regions (MRs) and hDMRs. (d) Overlap of hDMRs in all *et* mutants vs Col‐0 (WT).

The single‐strand cleavage function on DNA mediated by the GIY‐YIG domain in the N‐terminal half of the ET factors (Ivanov *et al*., 2008) suggested an effect on DNA methylation patterns analogous to described demethylases ROS1 and DME (Choi *et al*., 2002; Gong *et al*., 2002). Therefore, whole genome bisulphite sequencing (WGBS) of Col‐0, *et1‐1* and *et2‐3* single mutants and the *et1‐1 et2‐3* double mutant was performed. As ET factors show their maximum of expression in reproductive tissues (Ivanov *et al*., 2008), the analysis was focused on flower buds (12c–14 stage; Smyth *et al*., 1990). Principal component analysis (PCA) of differential DNA methylation showed clear separation of the genotypes and high similarity between biological replicates (Fig. 2a). Methylated regions (MRs) were identified in every sample using a previously published algorithm (Hagmann *et al*., 2015). Comparisons of Col‐0 and mutant lines revealed 352 highly differentially methylated regions (hDMRs) for *et1‐1*, 373 for *et2‐3* and 275 for the double mutant (see Table S2 for a list of hDMRs:). Cluster analysis of the hDMRs revealed preferential loss of methylation in the mutants compared to Col‐0 (Fig. 2b), mainly in the symmetric CG context (see Fig. S2a). Although genomic regions covered by hDMRs coincided mainly with transposable elements (TEs), hDMRs were proportionally over‐represented 2 kb upstream and 2 kb downstream of protein‐coding sequences (Fig. 2c). Methylated regions that were classified as non‐DMRs showed minor variation in methylation, confirming the specificity of our algorithm (see Fig. S2d). Among identified hDMRs, *MPF* (hDMR686) was found, showing *et‐1‐1*‐specific hyper‐methylation. *MPF* (Methylated region near Flowering locus C, AT5G10140) was described as a marker region for loss of demethylation function (Penterman *et al*., 2007a; Zhai *et al*., 2008). To validate the identified hDMRs the available *et*‐T‐DNA insertion mutant lines *et1‐1*,* et2‐1* and *et2‐3* (Fig. 1a) were tested by clonal bisulphite sequencing analysis (Fig. S1). AT1G26400 (FAD‐Berberine‐binding protein), AT1G34245 (*EPF2*, Epidermal Protein Factor2) hypermethylated in *ros1* and *dme* mutants, and AtSN1 as a reference region for RdDM (Kuhlmann & Mette, 2012) were tested. At *AT1G26400* and *AT1G34245*, a significant increase of cytosine methylation was detectable for both alleles of the *et2* mutant (*et2‐1* and *et2‐3*). This increase was preferentially caused by an increase of methylation in the symmetric CG context.

The largest hDMR (hDMR180: 1159 bp) was located on chromosome 1 : 28515015, completely covering a HELITRON1 element (AT1TE93275); this locus was depleted of methylation in all three mutant lines. Demethylation of this region was characteristic for *nerd* mutant plants (Pontier *et al*., 2012). Loss of this GYF‐and zinc‐finger (CCCH‐type) domain‐containing protein function led to definition of a plant‐specific chromatin‐based RNA silencing pathway depending on RDR1/6. The second region defining the NERD pathway was *psORF* (AT5G35935). This region was also detected as hDMR750 in the *et* mutants.

The identified hDMRs in the *et1* and *et2* single mutants overlapped substantially (Fig. 2d), suggesting similar regulatory function of ET1 and ET2 at these shared loci. In turn, *c*. 50% of hDMRs were specific to either mutant, indicating an additional gene‐specific influence of either of the two factors on DNA methylation. Differential DNA methylation with respect to Col‐0 was similar in both mutants for a large fraction of hDMRs (Fig. 2c). In total, 70% of hDMRs showed the same directional methylation change. However, distinct roles of ET factors in DNA methylation were also apparent: 15% of *et1‐1* hDMRs were hypo‐methylated in *et1‐1* but hyper‐methylated in *et2‐3*, while 6% showed the opposite pattern, which suggests antagonistic roles of ET1 and ET2 for methylation of these loci.

For the vast majority of hDMRs, DNA methylation in the *et1‐1 et2‐3* double mutant either reflected the situation in one of the single mutants, or showed additive effects, corroborating the combination of overlapping and specific function of ET1 and ET2 that we had already derived from the hDMR overlap analysis. Intriguingly, a small subset of hDMRs that showed loss of methylation in either *et1‐1* or *et2‐3* did not show methylation changes in the double mutant, suggesting epistatic interaction of ET1 and ET2 at these loci.

**Figure 3:**
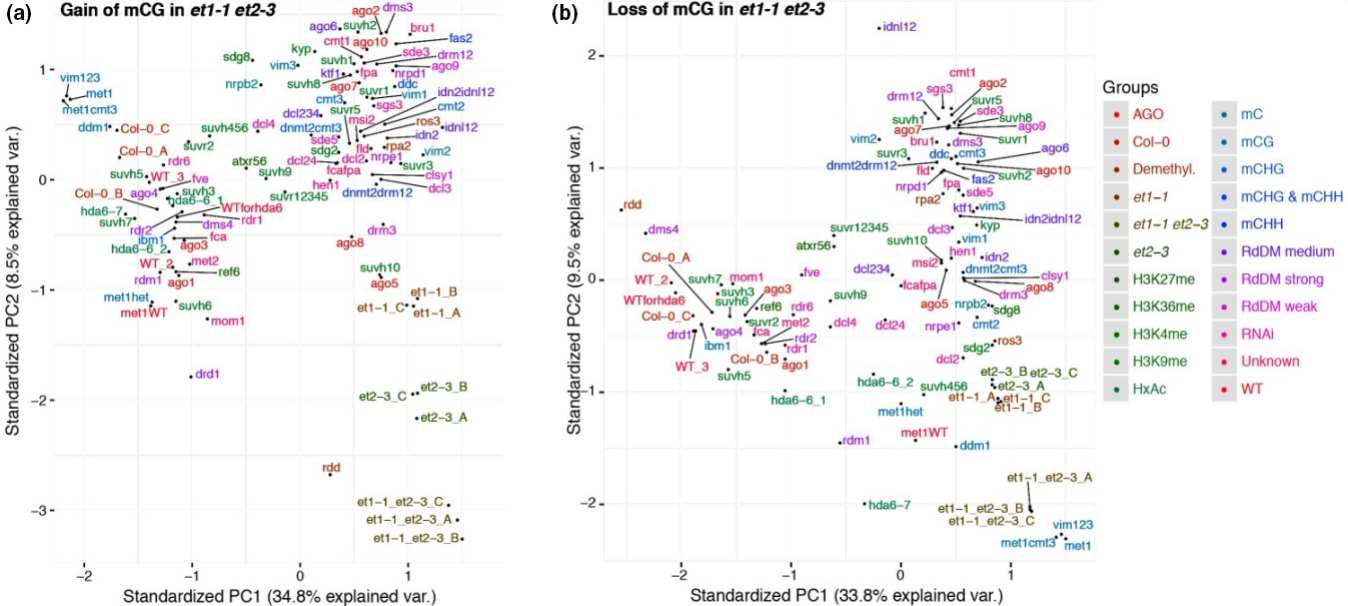
Principal component (PC) analysis of highly differential methylated regions (hDMRs) in *Arabidopsis thaliana et1‐1 et2‐3* vs Col‐0 (WT) and other epigenetic mutants (according Stroud *et al*., 2013). (a) hDMRs with gain of methylation in *et1‐1 et2‐3* of CG context. (b) hDMRs with loss of methylation in *et1‐1 et2‐3* of CG context.

To gain insights into the methylation pathway that ET1 and ET2 might be involved in, we next used the hDMR between Col‐0 and the *et1‐1 et2‐3* double mutant as a proxy to investigate DNA methylation at the same loci in a collection of previously published epigenetic mutants (Stroud *et al*., 2013). As CG methylation was the most prominently affected in *et1‐1 et2‐3*, we focused our analysis on this context. Analysis of hDMRs with gain of methylation in *et1‐1 et2‐3* revealed the closest similarity to methylation patterns of *rdd* mutant plants (Fig. 3a). *rdd* is a triple mutant defective for ROS1, DM2 and DML3 (Penterman *et al*., 2007b).

Regions which reduced CG methylation in *et1‐1 et2‐3* (Fig. 3b) compared to Col‐0 showed the closest similarity to *met1* (DNA METHYLTRANSFERASE 1; Kankel *et al*., 2003) and the triple mutant *vim1 vim2 vim3* (VARIANT IN METHYLATION; Shook & Richards, 2014), both defective for CG‐specific maintenance of methylation.

Although prior *in vitro* studies (Ivanov *et al*., 2012) showed that ETs bind to DNA irrespective of the sequence context, we investigated whether any sequence feature could be identified using the set of identified DMRs. Therefore, we choose the 136 hDMRs detected in *et2‐3* flower tissue which showed gain of methylation. We applied the motif‐based sequence analysis tool MEME (Bailey *et al*., 2006) on these potential ET2 DNA binding motifs. No motif could be identified, suggesting that the DNA binding is not sequence‐specific. The DIMONT approach (Grau *et al*., 2013), which includes sorting of the sequences according to intensities, did not reveal any binding pattern either. Importantly, when using a motif length of 10 (bgOrder = 0, motifOrder = 0, other parameters = default), we detected two adjacent pyrimidines (TT, CT, TC) as a recurring motif (Fig. S3).

### Transcriptome analysis to identify differentially expressed genes in *et* mutants

**Figure 4:**
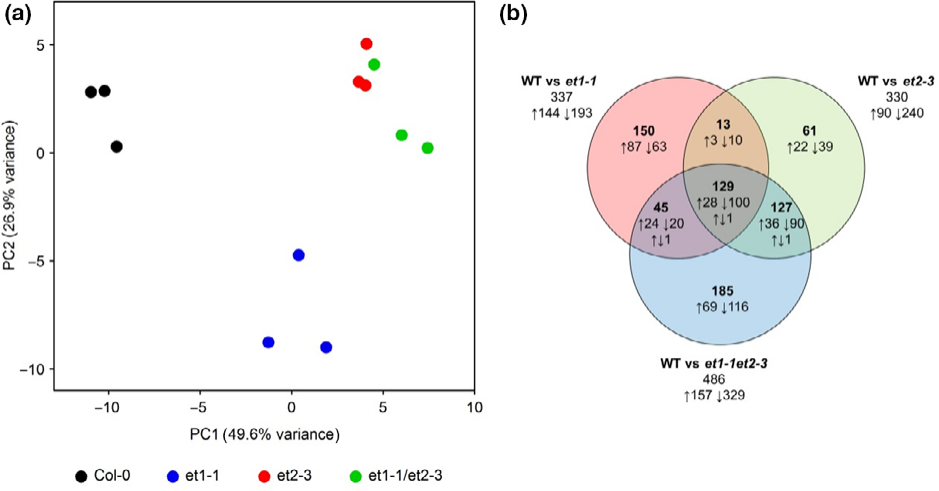
Differentially expressed genes (DEGs) in *Arabidopsis thaliana et* mutant flowers. (a) Principal component (PC) analysis of flower transcriptome. The plot shows the transcriptome data of *et1‐1*,* et2‐3* and *et1‐1 et2‐3* mutants and Col‐0 (WT) in triplicate. (b) Venn diagram of DEGs between Col‐0 (WT) and mutant flowers.

Although previous studies showed that a strong overlap of DMRs and differentially expressed genes (DEGs) cannot be expected (Havecker *et al*., 2012; Kawakatsu *et al*., 2016), we analysed the transcriptomes of flower buds from the same tissue used for WGBS. Triplicate strand‐specific cDNA libraries of Col‐0, *et1‐1*,* et2‐3* and the double mutant *et1‐1 et2‐3* yielded between 13.7 and 23.3 million short reads (107 nt), of which, after adapter and quality trimming, 7.2–12.3 million reads mapped in sense orientation onto annotated, nuclear gene models in the genome of *A. thaliana* (TAIR10, Table S3). PCA of normalised and mapped read counts revealed reliable separation of the mutant samples and showed that mRNA abundance of the double mutant was more similar to *et2‐3* than to *et1‐1* (Fig. 4a). DEGs were identified for pairwise comparisons between Col‐0 and *et* mutants. In total, 337, 330 and 486 DEGs with a false discovery rate (FDR) ≤ 0.01 and an absolute log_2_ fold change (lg2FC) ≥ 1 were found for the comparisons of Col‐0 vs *et1‐1*, Col‐0 vs *et2‐3*, and Col‐0 vs *et1‐1 et2‐3*, respectively (Fig. 4b). The number of transcripts downregulated in mutants (*et1‐1*, 193; *et2‐3*, 240; *et1‐1 et2‐3*, 329) was always larger than the number of upregulated ones (*et1‐1*, 144; *et2‐3*, 90; *et1‐1 et2‐3*, 157). Similar to hDMRs, we detected DEGs shared between the two single mutants (142) as well as DEGs private to either *et1‐1* or *et2‐3* (195 and 188, respectively), indicating partial functional redundancy of ET1 and ET2. The 185 transcripts differentially regulated in the *et1‐1 et2‐3* double mutant demonstrate that interactions between regulatory pathways influenced by ET1 and ET2 define gene sets not affected in the single mutants. With the exception of three genes, all transcripts affected in two (*et1‐1* and *et2‐3*, 142; *et1‐1* and *et1‐1*/*et2‐3*, 174; *et2‐3* and *et1‐1*/*et2‐3*, 256) or all three mutants (129) showed a consistent direction of change in the different lines. lg2FC values of the 129 transcripts significantly influenced in all three mutants showed that for 56 transcripts, the effects of *et1‐1* and *et2‐3* were additive, while for 72 transcripts the influence of one mutation was modulated by the other . This suggested epistatic interactions, similar to what we observed for DNA methylation effects.

### Functional analysis of DEGs

**Figure 5:**
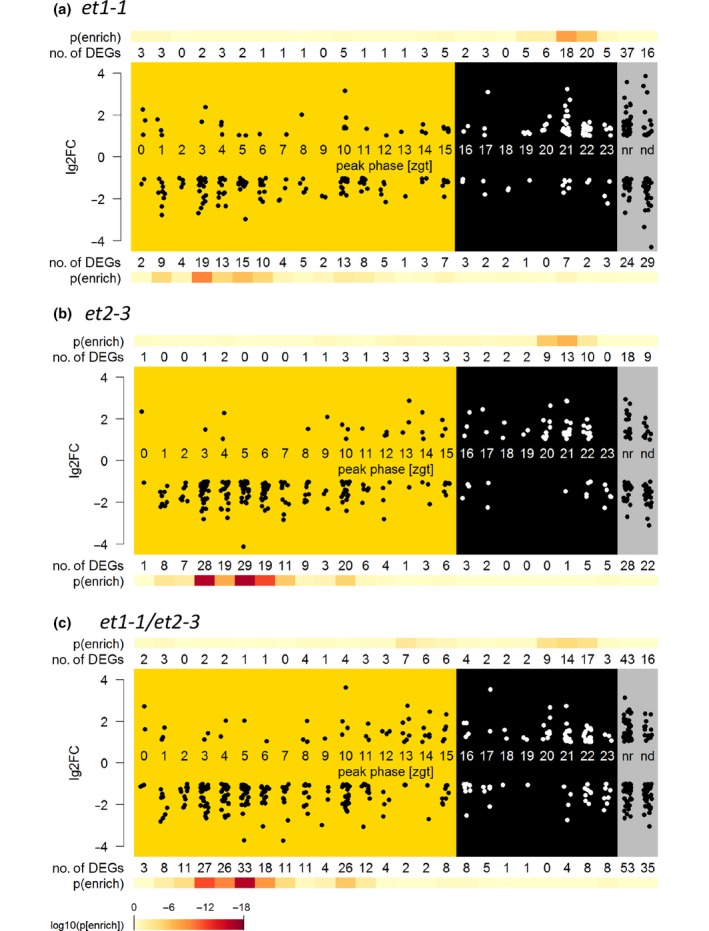
Differentially expressed genes (DEGs) in *Arabidopsis thaliana et* mutant flowers sorted according their peak phase of expression during the circadian rhythm for (a) *et1‐1*, (b) *et2‐3* and (c) *et1‐1/et2‐3*. Yellow areas, daytime (light); black areas, night phase (darkness); grey areas, genes which are nonrhythmic (nr) or not detected in a previous study based on the ATH1 chip (Mockler *et al*., 2007). Each point represent one DEG, and change is given in log_2_‐fold change (lg2FC). Colour intensity towards red indicates the level of significance of the number of DEGs for each zeitgeber (zgt) time‐phase.

A gene ontology (GO) term analysis for DEGs focusing on GOslim terms using bingo (Maere *et al*., 2005) showed that the terms ‘plastid’ and ‘thylakoid membrane’ in the category ‘cellular components’ were significantly enriched (FDR < 0.01) in all three *et* mutant comparisons against Col‐0 (Table 1). Also, the significant enriched terms in the category ‘biological process’ indicated that light‐regulated and light‐dependent photosynthesis was strongly affected in *et* mutants, even though there was no visible phenotype with respect to leaf colour and vitality. This result prompted us to inspect light‐regulated processes in greater detail, which led to the discovery that certain clock and flowering time genes were differentially expressed. Because care had been taken to harvest flower samples always at the same time of the long day light–dark cycle (3–5 h after lights came on), we were able to analyse DEGs in relation to their usual diurnal peak phase of expression (Mockler *et al*., 2007; Fig. 5a, *et1*; Fig. 5b, *et2*; Fig. 5c, *et1‐1 et2‐3*). DEGs with a peak phase in late night (zeitgeber time (ztg) 20–22) were generally overexpressed in *et* mutants, while DEGs with a peak phase during early morning (ztg 3–6) were generally downregulated. This indicated a delay of the clock phases in the *et* mutants.

**Table 1:** Enriched GOslim terms for genes differentially expressed in *et* mutant flowers

GOslim term	GO ID	*et1‐1* vs Col‐0			*et2‐3* vs Col‐0			*et1‐1 et2‐3* vs Col‐0		
		Genes	*P*_adj_		Genes	*P*_adj_		Genes	*P*_adj_	
Differentially expressed		337			330			486		
No GOslim annotation		6			3			6		
Cellular component										
Plastid	9536	48	1.90E‐04	*	56	3.40E‐07	*	73	3.20E‐07	*
Thylakoid	9579	12	3.10E‐03	*	16	1.80E‐05	*	21	2.90E‐06	*
Cell	5623	171	2.50E‐03	*	163	1.90E‐02		256	6.80E‐06	*
Cell wall	5618	22	1.00E‐06	*	11	8.10E‐02		22	1.40E‐04	*
External encapsulating structure	30312	22	1.00E‐06	*	11	8.10E‐02		22	1.40E‐04	*
Cytoplasm	5737	70	1.20E‐01		86	1.60E‐04	*	117	1.90E‐04	*
Extracellular region	5576	17	8.20E‐05	*	11	3.20E‐02		19	3.40E‐04	*
Membrane	16 020	67	2.50E‐03	*	57	8.40E‐02		95	3.40E‐04	*
Intracellular	5622	93	5.10E‐01		112	3.80E‐03	*	157	2.80E‐03	*
Peroxisome	5777	6	2.90E‐02		7	8.70E‐03	*	7	4.20E‐02	
Molecular function										
Catalytic activity	3824	133	4.20E‐06	*	135	5.60E‐07	*	205	9.90E‐12	*
Oxygen binding	19 825	9	8.20E‐03	*	12	1.30E‐04	*	17	4.10E‐06	*
Biological process										
Response to abiotic stimulus	9628	42	1.90E‐08	*	49	1.20E‐12	*	67	6.90E‐16	*
Response to stress	6950	51	9.50E‐07	*	55	7.40E‐09	*	73	1.30E‐09	*
Response to endogenous stimulus	9719	23	1.50E‐03	*	28	1.10E‐05	*	42	2.00E‐08	*
Secondary metabolic process	19 748	11	1.00E‐02		22	3.50E‐09	*	23	3.20E‐07	*
Cellular amino acid and derived metabolic process	6519	14	1.00E‐02		17	5.10E‐04	*	27	1.60E‐06	*
Metabolic process	8152	104	1.50E‐02		117	4.80E‐05	*	164	1.60E‐05	*
Carbohydrate metabolic process	5975	25	1.10E‐04	*	27	1.10E‐05	*	32	5.90E‐05	*
Photosynthesis	15 979	8	5.90E‐04	*	7	2.60E‐03	*	10	1.60E‐04	*
Response to biotic stimulus	9607	18	1.00E‐03	*	15	1.30E‐02		23	5.00E‐04	*
Catabolic process	9056	13	1.80E‐01		11	3.60E‐01		24	2.60E‐03	*
Response to external stimulus	9605	8	2.70E‐02		6	1.60E‐01		11	9.20E‐03	*

The BINGO app (Maere *et al*., 2005) of Cytoscape (Smoot *et al*., 2011) was used to determine enrichment for GOslim_Plant terms using the annotation of *Arabidopsis thaliana*. All enriched GOslim terms are given, for which an adjusted *P*‐value (*P*_adj_; Benjamini–Hochberg correction; *, *P *≤* *0.01) has been observed for differentially expressed genes in at least one comparison of *et* mutants vs Col‐0.

Among DEGs were several key regulators involved in the photoperiodic pathway (Table S3): *TIMING OF CAB EXPRESSION1* (*TOC1*), *CIRCADIAN CLOCK ASSOCIATED1* (*CCA1*), *LATE ELONGATED HYPOCOTYL* (*LHY*  ), *FLOWERING LOCUS T* (*FT*) and its homologous *TWIN SISTER OF FLOWERING LOCUS T* (*TSF*).

Based on the focus of our study, several genes were selected for individual inspection. This includes the *ET*‐gene family and the top 10 DEGs (Table 2). We inspected the top 10 DEGs for correlation of DNA methylation difference. hDMR and DMR lists from Table S2 were used, and the respective genes were additionally inspected for reproducible minor changes in DNA methylation among the triplicates (vDMR, visually detected differential methylated regions and single methylation polymorphisms (SMPs), visually detected single methylation polymorphisms). ET1 expression is lower than ET2 and ET2 shows a peak of expression in flower tissues. In the *et1‐1* T‐DNA insertion line as well as in the double mutant, 0–2 reads per million (RPM) are detected which were located downstream of the insertion site, confirming the absence of functional mRNA. Consequently *ET1* was found in the list of downregulated genes in *et1‐1*. An increase of DNA methylation upstream of the second intron associated with the *et1‐1* T‐DNA insertion was detectable (DMR1409). In the *et2‐3* mutant expression of *ET1* was not significantly different from that in Col‐0 (7–11 RPM).

**Table 2:** Top 10 list of differential expressed genes in *et* mutant flowers obtained by RNA sequencing

	*et1‐1*/Col‐0	DNA methylation	
AT4G34550	−4.297	SMP coding region	F‐box family protein
AT3G09450	−3.348	SMP coding region	Fusaric acid resistance protein (TAIR:AT2G28780.1)
AT1G02820	−2.971	No methylation	Late embryogenesis abundant 3 (LEA3) family protein
AT3G17609	−2.768	No methylation	HY5‐homologue
AT5G28030	−2.680	SMP coding region	L‐cysteine desulfhydrase 1
AT4G26170	−2.662	DMR1409 coding region	ET1 (TAIR:AT5G56780.1)
AT5G43630	−2.552	SMP coding region	Zinc knuckle (CCHC‐type) family protein
AT1G66725	−2.538	No methylation	MIR163; miRNA
AT2G21320	−2.447	No methylation	B‐box zinc finger family protein
AT3G02380	−2.362	SMP coding region	CONSTANS‐like 2
AT4G37800	2.536		Xyloglucan endotransglucosylase/hydrolase 7
AT3G62150	2.732	SMP	P‐glycoprotein 21
AT2G44460	2.819	vDMR coding, promotor	Beta glucosidase 28
AT5G05365	3.072	No methylation	Heavy metal transport/detoxification superfamily protein
AT1G65480	3.091	SMP promotor, incr. backgr	FT, PEBP family protein
AT5G48850	3.153	SMP coding region	Tetratricopeptide repeat (TPR)‐like superfamily protein
AT1G08930	3.229	vDMR 3′ region	Major facilitator superfamily protein
AT1G53480	3.371	vDMR coding	mto 1 responding down 1
AT4G20370	3.573	vDMR promotor	TSF, PEBP family protein
AT2G09187	3.851	hDMR243	Athila6 transposable element gene

	*et2‐3*/Col‐0	DNA methylation	
AT3G09450	−4.747	SMP coding region	Fusaric acid resistance protein (TAIR:AT2G28780.1)
AT4G34550	−4.519	SMP coding region	F‐box family protein (TAIR:AT2G16365.3)
AT1G02820	−4.125	No methylation	Late embryogenesis abundant 3 (LEA3) family protein
AT1G29920	−3.109	vDMR coding	Chlorophyll A/B‐binding protein 2
AT3G58990	−2.835	SMP 3′ region	Isopropylmalate isomerase 1
AT5G66300	−2.812	hDMR865 coding 5′	NAC domain containing protein 105, VND3
AT3G56290	−2.807		Unknown protein
AT3G48320	−2.797	SMPs coding region	Cytochrome P450, family 71, subfamily A, polypeptide 21
AT5G58770	−2.737	SMPs coding region	Undecaprenyl pyrophosphate synthetase family protein
AT3G13061	−2.696	SMPs coding region	Other RNA, put. nat. antisense RNA
AT1G15010	2.401		Unknown protein AT2G01300.1
AT1G65480	2.454	SMP promotor	FT, PEBP family protein
AT3G18550	2.615		TCP family transcription factor
AT1G56150	2.628	SMP coding region	SAUR‐like auxin‐responsive protein family
AT4G15690	2.722	No methylation	Thioredoxin superfamily protein
AT5G56780	2.835	SMP, DMR1890	Effector of transcription2
AT1G08930	2.871	vDMR 3′	Major facilitator superfamily protein
AT1G07050	2.874	No methylation	CCT motif family protein
AT5G65080	2.936	SMP 3′	K‐box region, MADS‐box transcription factor family protein
AT4G20370	3.160		TSF, PEBP family protein

	*et1‐1_et2‐3*/Col‐0	DNA methylation	
AT4G34550	−4.399	SMP coding region	F‐box family protein (TAIR:AT2G16365.3)
AT4G25470	−3.732	No methylation	C‐repeat/DRE binding factor 2
AT1G02820	−3.710	No methylation	Late embryogenesis abundant 3 (LEA3) family protein
AT2G42540	−3.065	vDMR promotor/5′	Cold‐regulated 15a
AT1G18330	−3.051		Homeodomain‐like superfamily protein
AT1G29920	−3.049	vDMR coding region	Chlorophyll A/B‐binding protein 2
AT5G52310	−2.984		Low‐temperature‐responsive protein 78 (LTI78/RD29A)
AT3G09450	−2.851	SMP coding region	Fusaric acid resistance protein(TAIR:AT2G28780.1)
AT2G31380	−2.803		Salt tolerance homologue
AT4G26170	−2.796	vDMR coding region	ET1 (TAIR:AT5G56780.1)
AT3G26210	2.557	SMP Promotor	Cytochrome P450, family 71, subfamily B, polypeptide 23
AT2G09187	2.568	hDMR243	Athila6 transposable element gene
AT1G56150	2.663	SMP coding region	SAUR‐like auxin‐responsive protein family
AT3G11340	2.710	vDMR coding region	UDP‐Glycosyltransferase superfamily protein
AT1G08930	2.719	vDMR 3′	Major facilitator superfamily protein
AT1G07050	2.742	No methylation	CCT motif family protein
AT3G57460	3.129	hDMR528 Promotor	Catalytics;metal ion binding
AT4G20370	3.415	vDMR promotor	TSF, PEBP family protein
AT1G65480	3.521	SMP promotor	FT, PEBP family protein
AT5G64120	3.612	vDMR coding region	Peroxidase superfamily protein

SMP: single methylation polymorphism, DMR: differential methylated region, vDMR: visual detected differential methylated region, hDMR: differential methylated region with high significance.

In contrast to the qPCR results, *ET2* was found in the top 10 lists of upregulated genes in *et2‐3* and the double mutant (lg2FC = 2.8). This might be caused by the integrated pROK2‐derived T‐DNA in the used SALK_151861 line leading to 35S promotor‐driven ectopic transcription (Daxinger *et al*., 2008). Inspection of reads and subsequent sequencing of the *et2‐3 ET2* gene revealed a 24 bp deletion at position 1203 in the third exon and confirmed the T‐DNA insertion located in the first exon 85 bp after the start ATG. A potential alternative translation start 869 bp after start ATG of the gene might lead to expression of a truncated version without DNA cleavage domain. Therefore, absence of functional full‐length *ET2* mRNA in the analysed *et2‐3* T‐DNA insertion plants could be confirmed. The differential expression was associated with the DMRs 1890 and 1891, located within the coding region of *ET2* showing reduction of methylation.

**Figure 6:**
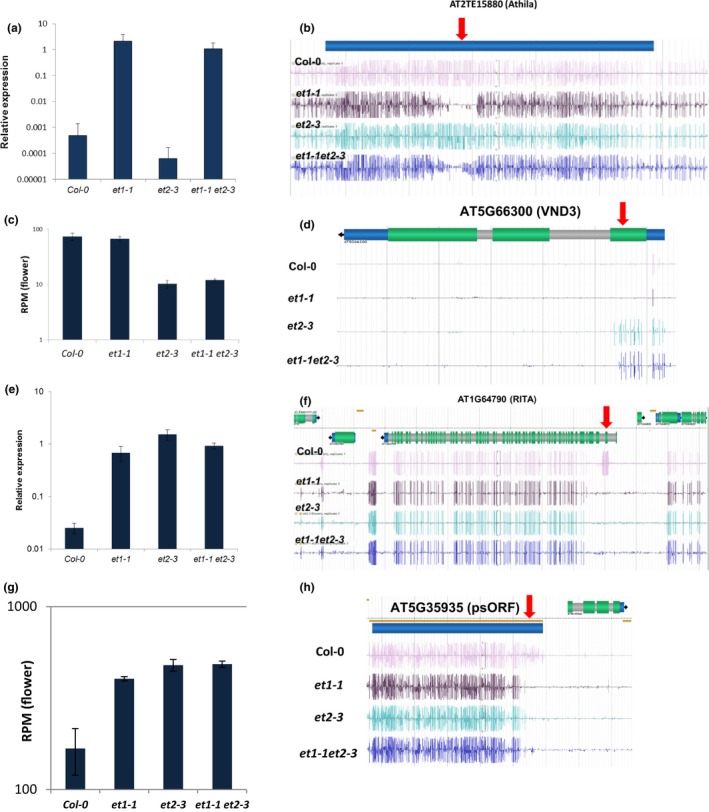
Selected differentially expressed genes (DEGs) in *Arabidopsis thaliana et* mutant flowers and correlation with differentially methylated regions (DMRs). (a) Relative expression of AT2G09187 (transcribed from AT2TE15880, Athila6A) confirmed by real‐time RT‐qPCR from shoot apical meristem (SAM). Bars indicate the mean of three independent samples with ± SE. (b) DNA methylation signature in the region of AT2TE15880 encoding the Athila6A retroelement. Red arrow indicates hDMR165. (c) Expression analysis of flower tissue of AT5G66300 (*VND3*) derived from RNA sequencing. Displayed are reads per million from three independent experiments. (d) DNA methylation signature in the region of AT5G66300 encoding VND3 with hDMR865 (red arrow). (e) Relative expression of AT1G64795 (RITA) confirmed by real‐time RT‐qPCR from SAM. (f) DNA methylation signature in the region of AT1G64790 annotated as ILYTHIA. AT1G64795 (RITA) transcripts are antisense orientated to ILYTHIA and covering hDMR153. Blue regions, untranslated regions; green regions, translated regions; grey regions, introns; red arrow points towards the respective hDMR865. Methylation signature shown is as a representative from three independent replicates. (g) Expression analysis of flower tissue of AT5G35935 (*psORF*) derived from RNA sequencing. Displayed are reads per million from three independent experiments. (h) DNA methylation signature in the region of AT5G35935 annotated as psORF; red arrow points towards the respective hDMR750.

The top upregulated gene in *et1‐1* and the *et1‐1 et2‐3* double mutant was the cDNA AT2G09187 (lg2FC = 6.54, *P*_adj_ = 1.1 × 10^−221^ in *et1‐1* and lg2FC = 6.52, *P*_adj_ = 9.7 × 10^−221^ in the double mutant), annotated as a transposable element gene and matching the annotated transposable element AT2TE15880 from the *Athila6A* family. We confirmed upregulation in the *et1‐1* mutant by qRT‐PCR (Fig. 6a). The induction is specific for the *et1‐1* mutant, indicating a functional difference between both ET mutants concerning the regulation of this transposon. The induced transcript overlapped with the highly differentially methylated region hDMR165 (Fig. 6b), with CG methylation loss specific to *et1‐1*.

The *top6* gene, which showed lg2FC = −2.8 higher transcript abundance in the *et2* and *et1‐1 et2‐3* flowers, is *VND3* (AT5G66300, Fig. 6c). VND3 is a VASCULAR‐RELATED NAC‐DOMAIN transcription factor (Yamaguchi *et al*., 2010; Zhou *et al*., 2014) associated with xylem vessel formation (Ivanov *et al*., 2008). Transcriptional suppression of *VND3* was associated with a gain of methylation (hDMR865) at the transcriptional start site of the gene (Fig. 6d).

One gene not present among the top 10 DEGs (sense), but being lg2FC = 4.2 up‐regulated in all mutants (Fig. 6e), was associated with hDMR153 (Fig. 6f). This region is referred to as RITA (AT1G64795, encoded in antisense orientation upstream of ILYTHIA, AT1G64790 and not in the TAIR10 dataset) already described as a metastable DMR (Havecker *et al*., 2012). As mentioned above, *psORF* (AT5G35935), hypomethylated in both *et* mutants (hDMR750), was found to be transcriptionally activated in the mutants (Fig. 6g,h).

In *et1‐1*, a complementation approach was performed using the endogenous *ProET1:ET1* sequence. For the majority of up‐regulated genes in *et1‐1,* namely AT5G48850 (*SDI1*), AT1G65480 (*FT*  ), AT2G44460 (*BGLU28*), AT4G31800 (*WRKY18*), AT5G40360 (*MYB115*), AT2G09187 (*Athila6A*) and the 5′ located antisense transcripts of AT1G64790 (ILLITHYA) RITA (Havecker *et al*., 2012), transcript level could not be restored to the Col‐0 level by transgenic insertion of *ProET1:ET1*. For genes found to be downregulated, such as AT1G26770 (*EXPA10*), AT1G02820 (*LEA*) and AT4G27330 (*SPL*) restoration of *ET1* transcript level resulted in Col‐0‐like expression (Fig. S4).

### Phenotypic characterisation of mutants

The phenotypic analysis of *et* mutants revealed a series of pleiotropic anomalies during plant development, similar to many other epigenetic pathway mutants (Kakutani *et al*., 1996; Ronemus *et al*., 1996).

**Figure 7:**
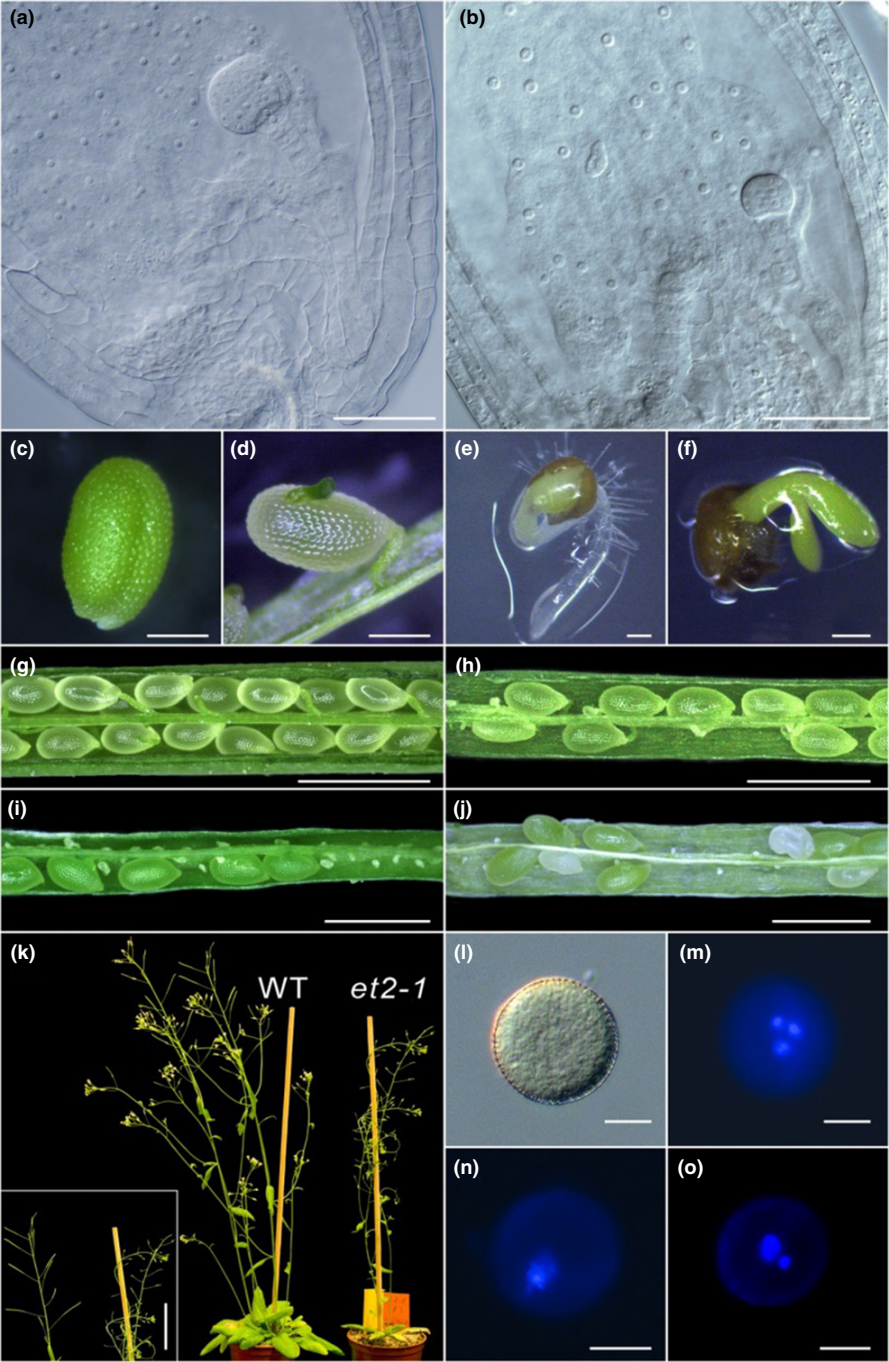
Phenotypic characterisation of various reproductive organs. Affected endosperm differentiation in *et* mutants. In comparison to *Arabidopsis thaliana* Col‐0 (a) the endosperm nuclei are greatly enlarged in the *et1‐1/et2‐1* double mutant. (b) The morphological effect has been quantified in all *et* mutants (Supporting Information Fig. S5a). Precocious germination of *et* mutants in siliques. (c) Col‐0 immature seed. (d) *et2‐3* immature seed germinates as early as in the siliques with the cotyledon permeating first along the side of the seed coat. Precocious germination *in vitro*. In Col‐0 seed the radicle penetrates the seed coat first (e), while in the *et1‐1* mutant (f) the cotyledon penetrates first. The *in vitro* germination was quantified for all *et* mutants (Fig. S5b). Dissected Col‐0 silique with viable seeds at late walking stick embryo stage (g) and *et* mutant siliques at a comparable developmental stage containing infertile ovules of (h) *et1‐1*, (i) *et2‐1* and (j) *et1‐1/et2‐1*. The quantification of infertile ovules observed in siliques of Col‐0 and *et* mutants is reported in Fig. S5(g). (k) Comparison between a Col‐0 plant (left) and an *et2‐1* plant (right) shows high sterility in the *et* mutant. In the left corner a detail of the sterile siliques is shown. (l–o) Distortion in *et* mutant pollen. Col‐0 pollen under (l) bright‐field imaging and (m) after DAPI‐staining: Col‐0 pollen nuclei show the larger vegetative nucleus and two generative nuclei. Various distortions during pollen differentiation were found in *et* mutants. (n) Completely collapsed pollen in *et1‐1* and (o) pollen with only one generative nucleus in *et2‐3*. Complete quantification of pollen nuclei distortion is reported in Fig. S5(e). Bars: (a, b) 50 μm; (c–f) 0.2 mm; (g–j) 1 mm; (k) 3 cm; (l–o) 10 μm.

One of the phenotypic defects observed in *et* mutant plants became apparent during endosperm differentiation. The endosperm nuclei of the *et* mutants exhibited a characteristically altered morphology with greatly enlarged nucleoli, possibly indicating enhanced transcriptional activity of rDNA genes (Shaw & Brown, 2012; Baker, 2013) or activated DNA damage repair (Kobayashi, 2008; Shaw & Brown, 2012) (Fig. 7a,b). In contrast to Col‐0 seeds (Fig. 7a), *et1‐1* and *et2‐3* showed enlarged nuclei in *c*. 25% of the samples analysed via differential interference contrast (DIC) microscopy (*n* = 250 seeds; *n* = 65, *n* = 68, respectively). In the single mutant *et2‐1*,* c*. 30% of endosperms exhibited enlarged nucleoli (*n* = 75), and in the double mutant *et1‐1 et2‐1*, up to 70% of endosperms were found with increased nucleoli (*n* = 172; Figs 7b, S5).

In *et* mutants immature seeds started to germinate as early as in the silique. While during regular germination the radicle penetrates the seed coat first, in *et* mutants the cotyledon emerged first along the side of the seed coat (Fig. 7c,d). No precocious germination was detected in the Col‐0 control. Precocious germination of the *et* mutants was also observed when immature seeds were germinated *in vitro* (Fig. 7e,f), and the *in vitro* germination rate of mutants was elevated (Fig. S6).

The male gametophytes, which develop within the anther, consist of two sperm cells encased within a vegetative cell. Pollen of Col‐0 and *et* mutants was analysed using DAPI staining (*n* = 200 each line). In Col‐0, the vegetative nucleus and the two generative nuclei were clearly distinguishable, whereas many abnormal and collapsed pollen grains were detectable in *et* mutants (Figs 7l–o, S7). The female gametophyte or embryo sac develops within the ovule and consists of two synergids, one egg cell, one central cell and three antipodal cells which degenerate at the mature stage before fertilization (Drews & Koltunow, 2011). In the *et* mutants, the fusion of the two polar nuclei was partially compromised. The single mutants *et1‐1* and *et2‐3* showed *c*. 10% female gametophytes with distortions of polar nuclei fusion (*n* = 40 and *n* = 45, respectively), *et2‐1 c*. 5% (*n* = 24) and *et1‐1 et2‐1 c*. 15% (*n* = 63; Fig. S8).

The *et* single and double mutant plants also exhibited reduced fertility. Seed set studies of five plants (10 siliques per plant) revealed in *et1‐1* and *et2‐3* mutants that 10% and 20% of ovules were infertile, respectively. In the *et2‐1* mutant as well as in the double mutant *et1‐1 et2‐1*, a striking 60% of ovules were infertile (Fig. 7g–k).

**Figure 8:**
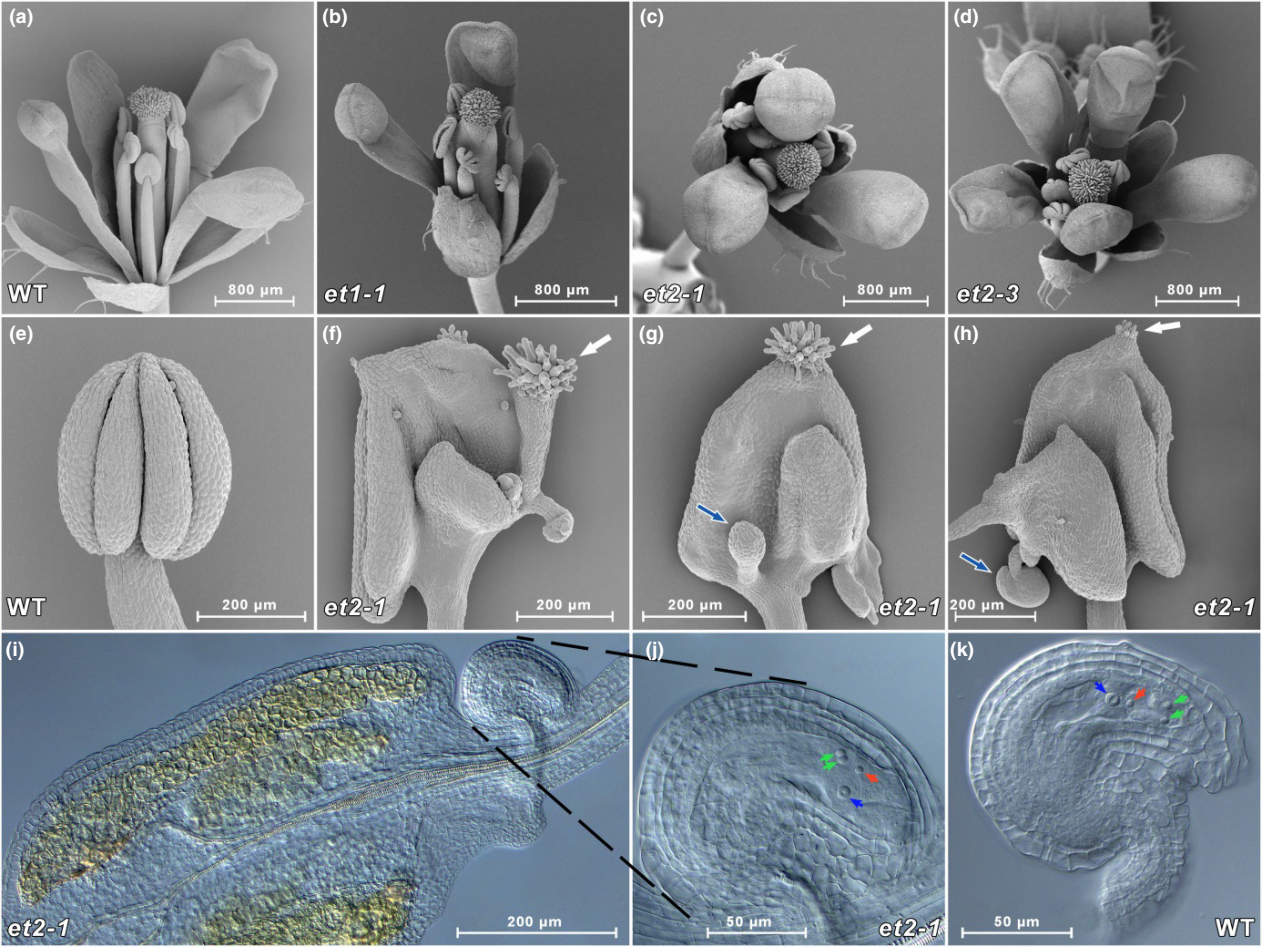
Phenotype of flower organs and stamen‐derived ovules. Changed numbers of flower organs in *Arabidopsis thaliana et1‐1*,* et2‐1* and *et2‐3* mutants and homeotic transformation of anthers into carpel‐like structures in *et2‐1* mutants. Reflection electron micrographs (REM) of (a) Col‐0 (WT), and (b) selected mutant flowers with two petals and two sepals in *et1‐1*, (c) with three petals in *et2‐1*, and (d) five petals in *et2‐3*. Reflection electron micrographs of (e) Col‐0 anthers and (f–h) various homeotic transformations of anthers into carpel‐like structures including stigma (white arrows) and ovule formation in *et2‐1* mutant plants (blue arrows). (i, j) Stamen‐derived ovules of the *et2‐1* mutant containing a fully developed gametophyte with egg cell (red arrows), two synergid cells (green arrows) and central cell (blue arrows). The normal polarity of the gametophytic cell types is partially distorted in (j) *et2‐1* ovules when compared with (k) Col‐0 ovule. Quantification of the altered flower organ number and of the homeotic phenotype is reported in Supporting Information Fig. S5(c,d).

The flowers of single and double *et* mutants had aberrant organ numbers, with all whorls being affected (Fig. 8). Flower morphology was quantified from 180 flowers from eight plants per genotype. In *et1‐1*, 20% of the sepals, 60% of the stamens and 30% of the petals showed anomalies. In *et2‐3*, 10% of the sepals, 65% of the stamens and 10% of the petals displayed defects (Fig. S9). Homeotic transformations were also found. The most frequent transformation was the conversion of the stamen into carpel‐like structures (Fig. 8). The *et2‐1* mutant showed a homeotic transformation rate of *c*. 50%, whereas the double mutant *et1‐1 et2‐1* reached *c*. 80%. The double mutant showed formation of multiple ovules (up to five per transformed anther) and several stigma‐like structures (up to four per transformed anther) (Fig. S9). To characterise the stamen‐derived ovules in more detail, the tissue was cleared and analysed by DIC microscopy. The ectopic ovules contained a normal gametophyte with fully developed egg cell, synergids and central cell. The normal polarity (synergids localized next to the micropyle, followed by egg cell and central cell) was distorted in the ectopic ovules (Fig. 8).

### ET2 mutant plants accumulate mutations

Based on our hypothesis and supported by the results obtained from phenotypic inspection (enlarged nucleoli, pleiotropic phenotypes), methylation (DMRs) and transcriptional (flowering time, delay in circadian rhythm) analyses, we hypothesised that ET factors are involved in DNA damage repair.

**Figure 9:**
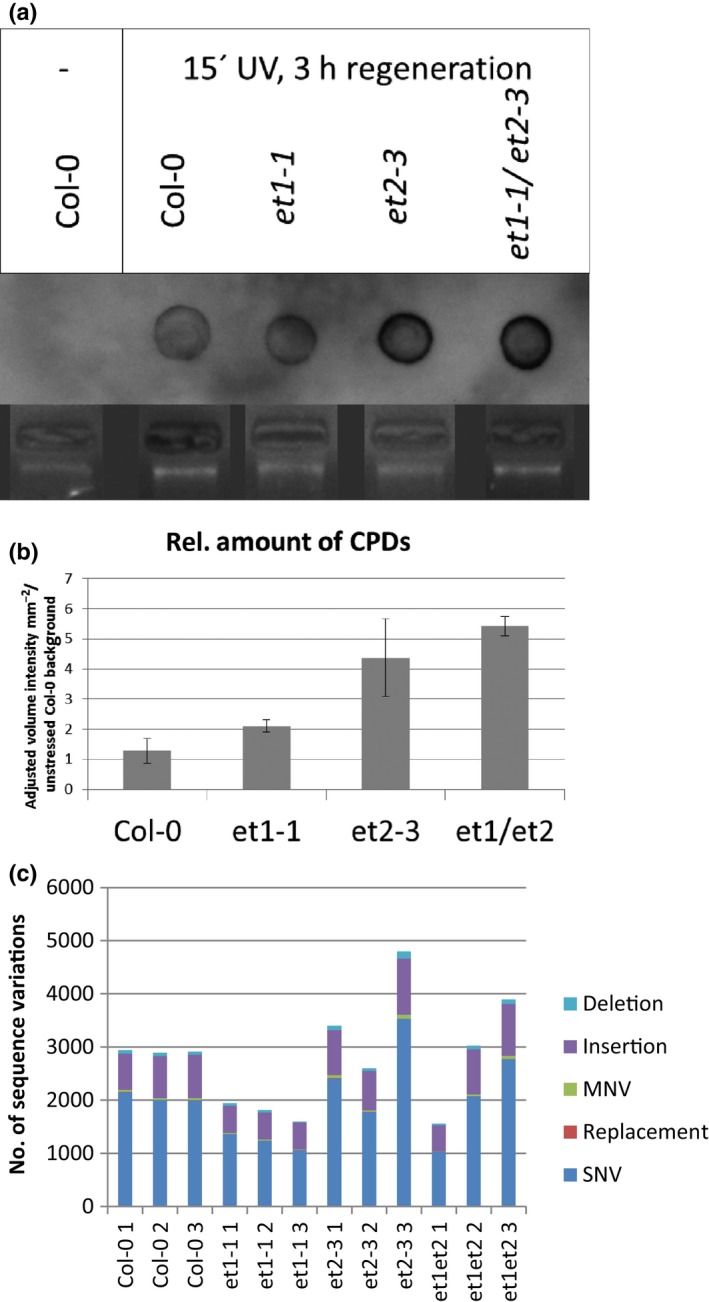
Higher mutation rate in *Arabidopsis thaliana et2* mutant plants. (a) Immunodetection of cyclobutane pyrimidine dimers (CPDs) on dot blotted genomic DNA (bottom) extracted from plants after 15 min of UV stress and 3 h regeneration time using a primary antibody against thymidine dimers. (b) Quantification of CPD immunosignals: CPDs were quantified as mean of relative amount of adjusted volume intensity mm^−2^ relative to untreated Col‐0 background signal. SE indicates the variation among three independent experiments. (c) Sequence variation analysis of *et* mutants using the RNA sequencing dataset. Colours indicate type of sequence variant.

For this analysis 1‐wk‐old seedlings were actively stressed by high‐intensity UV light. The UV‐induced CPDs were analysed by immunodetection (Moriel‐Carretero & Aguilera, 2010). While no CPDs were detectable in untreated control samples (Fig. S10), a clear signal was observed for UV‐treated Col‐0 (Fig. 9a). Slight quantitative differences were detectable in stressed *et1‐1*, whereas *et2‐3* and *et1‐1 et2‐3* showed a strong qualitative increase of detectable CPDs after UV stress (Fig. 9b).

We used the RNA‐seq data to search for new mutations compared to the *A. thaliana* reference sequence (TAIR10). Although this approach is hampered by the fact that RNA editing appears as sequence variations (Shao *et al*., 2014), this method was chosen to quantify differences. Detectable sequence variation can be classified into deletions, insertions, multinucleotide variations (MNVs), replacements and single nucleotide variations (SNVs). The group of SNVs is the most abundant class of mutations. Compared to the reference genome, a similar number of SNVs were detected among the samples in the Col‐0 reference plants. Interestingly, the number of detectable SNVs in the *et1‐1* plants was slightly lower, indicating a closer similarity to the TAIR10 reference genome, probably due to the Col‐0 initially used for generation of the *ET‐1* T‐DNA insertion. In the *et2‐3* plants a stronger deviation between the tested samples was recognised, with the highest accumulation of SNVs in *et2‐3* sample 3. A similar variation was detected in the *et1‐1/et2‐3* samples (Fig. 9c).

## Discussion

### Identified DMRs in the *et* mutants indicate similarities to mutants with impaired demethylation

Based on our hypothesis that ET factors act on DNA by single‐strand cleavage, gain of methylation is expected for ET‐target regions in the ET loss of function mutants. Therefore, identified DMRs were separated for gain and loss of methylation and analysed individually. Approximately one‐third of the identified hDMRs showed gain of methylation in the mutants and two‐thirds loss of methylation. The detectable hypomethylation might result from complex feedback regulation, also reported for *ros1* (Zhu *et al*., 2007) and *dme* mutant plants (Ortega‐Galisteo *et al*., 2008). The evolutionary neo‐functionalisation of the ET2‐based gene duplication might explain the identified ET1‐ and ET2‐specific DMR and DEGs.

PCA of hypomethylated hDMRs in *et* mutants indicated similarities with *met1* and *vim123* mutants (Kim *et al*., 2014; Shook & Richards, 2014). This observation indicated the unspecific loss of methylation in genomic regions which are under control of the DNA methylation maintenance pathway. The hypermethylated hDMRs exhibit a high similarity to DMRs detected in the genome of the *rdd* mutant, a triple mutant defective for ROS1 and DeMeter Like‐2 and 3 (DML2 and DML3) (Penterman *et al*., 2007b), all involved in demethylation of DNA. Moreover, selected regions known to be affected by active demethylation such as AT1G26400, AT1G34245 and AT5G10140 were found to be hypermethylated in all sequence contexts in the *et* mutants. Together this implies either a function of ETs in demethylation via deregulating ROS1, DME and DML2/3 or ETs represent another novel component of the active demethylation pathway. Because *DME* and *DMLs* are not found among the differentially expressed genes in *et* mutants, we favour the second view. As regulation at the post‐translational level or upregulation in a different context cannot be excluded, the detailed molecular mechanism remains to be investigated.

### Rare overlap between DEGs and regions with strong methylation difference (hDMRs)

Using our definition of hDMRs and DEGs, an overlap of regions and expression was barely detectable: AT2G09187 (overlapping with the Athila6A retrotransposon AT2TE15880) with hDMR165, AT5G56780 (*AtET2*) with hDMR517 and AT5G66300 (*VND3*) overlapping with hDMR865. Such rare overlap between DMRs and DEGs has been reported by several other studies (Havecker *et al*., 2012; Kawakatsu *et al*., 2016). The position of the DMR, located in a gene body or promotor, together with its genomic environment make it difficult to predict whether the change in methylation is a cause or consequence of differential expression. The rare overlap might indicate that our criteria defining an hDMR definition are very strict. As shown in the top list of DEGs, there are small regions of differential methylation as well as a number of SMPs in the genomic environment of DEGs (Table 2). These might lead to a difference in the amount of detectable transcripts of the respective genes in the mutants.

The over‐representation of detectable hDMRs associated with coding regions indicates a mechanism which might be associated with histone modifications such as H3K9 acetylation (H3K9ac) and H3K4 trimethylation (H3K4me3) (Ha *et al*., 2011), and these chromatin marks might also improve the recognition of putative ET binding sites. Such influence has been described for the histone acetyltransferase IDM1 which is required for demethylation (Qian *et al*., 2012). In addition, the identification of the NERD‐pathway target genes (Pontier *et al*., 2012) HELITRON1 (AT1TE93275) and *psORF* (AT5G35935) might indicate an association of H3K4 histone modification with ET function.

### Specific cases of metastable DMRs and their associated change in gene expression

Our studies identified two genic regions with a strong correlation between differential methylation and associated gene expression in the *et* mutants. AT1G64795 (*RITA*) was described as a metastable DMR (Havecker *et al*., 2012). The identified DNA methylation pattern was found to correspond to Col‐0 6000, which was the corresponding genotype used for the SALK T‐DNA collection. Although expression of this region was not reduced by transgenic reconstitution of ET1 function in the *et1‐1* mutant, we cannot conclude whether ETs affect this metastable locus or whether the identified loss of methylation is derived from the use of Col‐0 6000 as SALK mutant background.

The second region, specific for *et1‐1*, corresponds to the *Athila6* transposon belonging to the clade of the *Ty3/Gypsy* family (Pelissier *et al*., 1995; Wicker *et al*., 2007; Slotkin, 2010). Transcriptional activation of *Athila6A* was reported for mutants of the *MOM1* gene encoding a regulator of transcriptional gene silencing (Yokthongwattana *et al*., 2010), for mutants of the gene *MORC6* which is required for heterochromatin condensation and gene silencing (Moissiard *et al*., 2014), and for mutants of *ARABIDOPSIS TRITHORAX RELATED PROTEIN ATX5* and *ATX6* (Jacob *et al*., 2014).

Similar to *RITA*, the expression level of *Athila6* was not reduced by transgenic reconstitution of ET1 function. Again, we could not determine whether loss of ET function mediates a heritable, noncomplementable modification.

### Indications for a function of ET factors in DNA repair

The transcriptional induction of ET1 by ionising radiation (Culligan *et al*., 2006) supported the initial hypothesis (Ivanov *et al*., 2008) that ETs are involved in DNA repair mechanisms. Inspection of the genevestigator database (Zimmermann *et al*., 2004) identified the nucleoside antagonist Cordycepin (3′‐deoxyadenosine) as the strongest inducer of *ET2* transcription (Fig. S11), also reported to affect the DNA damage response (Lee *et al*., 2012).

The enlarged nuclei detected in the endosperm of *et* mutants indicates activated DNA damage repair (Kobayashi, 2008; Shaw & Brown, 2012). Activation of retroelements as detected in particular for the *et1‐1* mutant might cause subsequent mutations. Also, the enhanced DNA damage can delay expression of circadian genes (Chung *et al*., 2016), which in turn might affect DNA methylation patterns and expression of downstream target genes (Chow & Ng, 2017).

The reduced expression of AT4G27330 (*SPL*,* SPOROCYTELESS*; Yang *et al*., 1999) is a molecular feature associated with the precocious germination and incorrect orientation of the female gametophyte resulting in germination with cotyledons first. In addition, incorrect organisation of organ number and failures in development observed in correlation with the reduced expression found in *et1‐1* and *et2‐3* resemble the described phenotypes based on SPL reduction (Ito *et al*., 2004; Liu *et al*., 2009). The misexpression and phenotypic prominences could be complemented by expression of *ET1* arguing for a direct effect of ET function. A stress‐sensing mechanism including DNA damage regulating SPL expression has already been discussed (Zhao *et al*., 2017).

Here we show the accumulation of CPDs in *et* mutants after UV stress. As the loss of ET function resulted in an accumulation of unrepaired thymidine dimers, we propose that ET factors are involved in the mechanism of DNA repair. A similar observation was reported for *ros1* and *ddm1* (Questa *et al*., 2013). ROS1 acts as glycosylase and loss of its function also results in hyper‐methylation of specific genomic regions (Morales‐Ruiz *et al*., 2006). PCA of hyper‐methylated hDMRs, representing ET sites of action, revealed a close similarity to *rdd* mutants, which are also affected by a disturbed DNA damage repair mechanism. Close inspection of hDMRs in the ET2 mutant revealed no specific binding sequence, but suggested the presence of two adjacent pyrimidine nucleotides. It was reported that methylated cytosines are more susceptible to UV‐induced CPD formation (Martinez‐Fernandez *et al*., 2017). Based on the structure and *in planta* phenotypes we propose that ETs bind at DNA regions including CPDs with preferential histone modification. Here ETs might act by DNA cleavage and by initiating DNA repair.

The identification of clock‐related DEGs using GO analysis further suggests the DNA repair mechanism. The interplay of clock genes and UV‐B response has already been described (Sancar *et al*., 2000; Thompson & Sancar, 2002; Horak & Farre, 2015). The detected delay in the circadian rhythm in *et* mutants is in agreements with CRY1‐related repair mechanisms (Sancar *et al*., 2000; Thompson & Sancar, 2002). The differential expression of HYH (*et1‐1*), a key regulator of the UV‐B response (Binkert *et al*., 2014), and the linker Histone1‐3 (AT2G18050) supports the proposed function of ETs. H1‐3 is required for stress adaption on the chromatin level (Rutowicz *et al*., 2015). RNA interference directed against *H1‐3* affected the imprinting mechanism and DNA methylation (Rea *et al*., 2012). A search for DME downstream targets by analysis of DME overexpression revealed strong upregulation of *H1‐3* (Ohr *et al*., 2007). Therefore, downregulation of *H1‐3* might also contribute to detection of ET‐mediated DNA methylation differences.

The increased number of detectable SNPs in *et2‐3*, derived from the analysis of RNA sequencing data, supports the idea that ET2 acts in DNA repair. The detectable pleiotropic phenotypes, which occur at random and are heritable, might result from a higher mutation rate as a consequence of reduced DNA damage repair. In summary, all indications point toward a role of ETs as novel factors involved in DNA methylation in *A. thaliana*.

## Author contributions

HB conceived the project; FT, PR, BTMH, AC, TR, MK and CB performed the research; MK, FT, LA, CB, SS, IG, DW and HB analysed the data. MK, FT, CB and HB wrote the article with contributions of all the authors.

## Supporting information

Please note: Wiley Blackwell are not responsible for the content or functionality of any Supporting Information supplied by the authors. Any queries (other than missing material) should be directed to the *New Phytologist* Central Office.

**Fig. S1** DNA methylation of selected regions analysed in detail by bisulphite sequencing (%).
**Fig. S2** Cluster analysis of *et* mutant hDMRs relative to Col‐0 methylation.
**Fig. S3** Identification of common motifs in *et2‐3* highly differential methylated regions (hDMRs).
**Fig. S4** qPCR analysis of ET1 complementation.
**Fig. S5** Affected endosperm differentiation in *et* mutants.
**Fig. S6** Precocious germination of *et* mutants.
**Fig. S7** Quantification of pollen nuclei distortion.
**Fig. S8** Distorted embryo sac development in *et* mutants.
**Fig. S9** Homoeotic transformation of stamen into carpel‐like structures in double mutant plants.
**Fig. S10** Immunodetection of CPDs on dot blotted genomic DNA extracted from leaf tissue of 2‐wk‐old plants.
**Fig. S11** Genevestigator analysis of ET‐gene expression.
**Methods S1** Detailed information on RNA deep sequencing, DNA methylation analysis and Microscopy techniques.

**Table S1** Primers used in this study.

**Table S2** hDMRs and DMRs.

**Table S3** DEGs.

## Data Availability

Results of the whole genome bisulphite sequencing have been deposited at the European Nucleotide Archive under accession number PRJEB12413. DNA methylation data have been uploaded to the epigenome browser of the EPIC Consortium (https://www.plant-epigenome.org/; https://genomevolution.org/wiki/index.php/EPIC-CoGe) and can be accessed at http://genomevolution.org/r/939v. The flower and seedlings transcriptome data have been deposited at the European Nucleotide Archive under accession numbers PRJEB19779 and PRJEB14889, respectively. All data deposited will be made publicly available upon publication.
