# Supplementary material for: EFFECTOR OF TRANSCRIPTION factors are novel plant‐specific regulators associated with genomic DNA methylation in Arabidopsis
Source: New Phytol. 2018 Sep 25;221(1):261–78. doi: 10.1111/nph.15439 (PMC6585611; doi:10.1111/nph.15439)
Supplement: Supplementary file 1 — Fig. S1 DNA methylation of selected regions analysed in detail by bisulphite sequencing (%). Fig. S2 Cluster analysis of et mutant hDMRs relative to Col‐0 methylation. Fig. S3 Identification of common motifs in et2‐3 highly differential methylated regions (hDMRs). Fig. S4 qPCR analysis of ET1 complementation. Fig. S5 Affected endosperm differentiation in et mutants. Fig. S6 Precocious germination of et mutants. Fig. S7 Quantification of pollen nuclei distortion. Fig. S8 Distorted embryo sac development in et mutants. Fig. S9 Homoeotic transformation of stamen into carpel‐like structures in double mutant plants. Fig. S10 Immunodetection of CPDs on dot blotted genomic DNA extracted from leaf tissue of 2‐wk‐old plants. Fig. S11 Genevestigator analysis of ET‐gene expression. Methods S1 Detailed information on RNA deep sequencing, DNA methylation analysis and Microscopy techniques. [file NPH-221-261-s001.pdf]

New Phytologist Supporting Information

Figs S1–S11 and Methods S1

Article title:

EFFECTOR OF TRANSCRIPTION factors are novel plant-specific regulators associated with genomic DNA methylation in Arabidopsis

Authors: Francesca Tedeschi, Paride Rizzo, Bui Thi Mai Huong, Andreas Czihal, Twan Rutten, Lothar Altschmied, Sarah Scharfenberg, Ivo Grosse, Claude Becker, Detlef Weigel, Helmut Bäumlein and Markus Kuhlmann

Article acceptance date: 1 July 2018

Figure S1

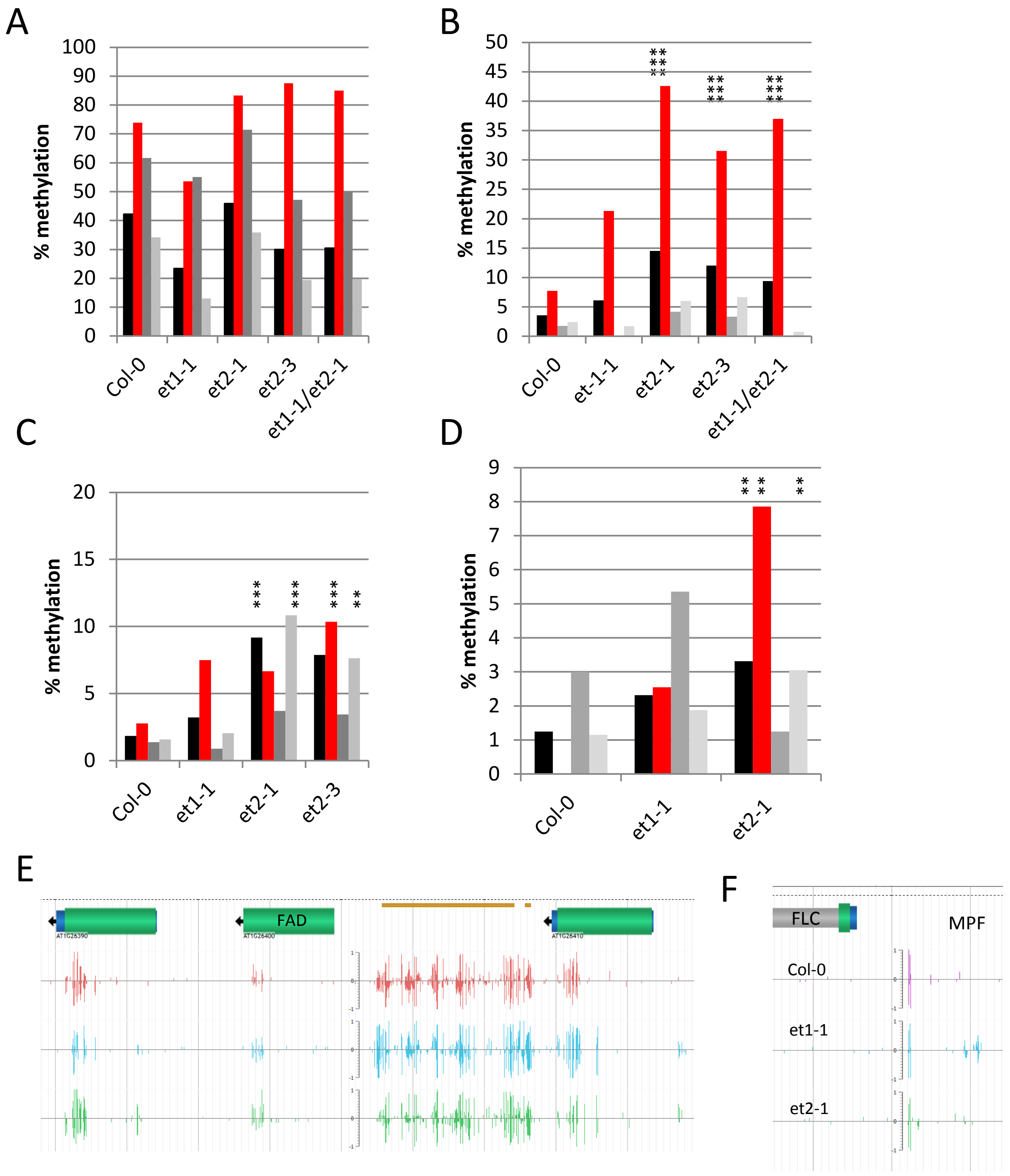

DNA methylation of selected region analysed in detail by bisulfite sequencing [%].

N indicates the number of clones sequenced per target and genotype. Stars indicate significant difference to Col-0: ***<0,005, **<0,01 by Chi square test. Cumulative methylation levels at all cytosines in the analysed region (black columns), cytosines in CG context (red columns), CHG context (grey columns; H stands for A, C or T) and CHH context (bright grey columns) are given in %.

A. Facultative heterochromatic AtSN1: N=22 (Col-0), 7(*et1-1*), 6(*et2-1*), 10(*et2-3*), 10(*et1-1/et2-1*).

B. AT1G26400 (FAD-Berberine-binding protein): N=19 (Col-0), 14 (*et1-1*), 18(*et2-1*), 15 (*et2-3*), 11 (*et1-1/et2-1*);

C. AT1G34245 (EPF2): N= 36(Col-0), 56 (et1-1), 27 (et2-1), 29 (et2-3)

D. MPF (methylated region near FLC, AT5G10140), N= 25 (Col-0), 28 (et1-1), 20 (et2-1)

E. Browser view for corresponding regions AT1G26400 and F . MPF, methylated region near FLC, AT5G10140

Figure S2

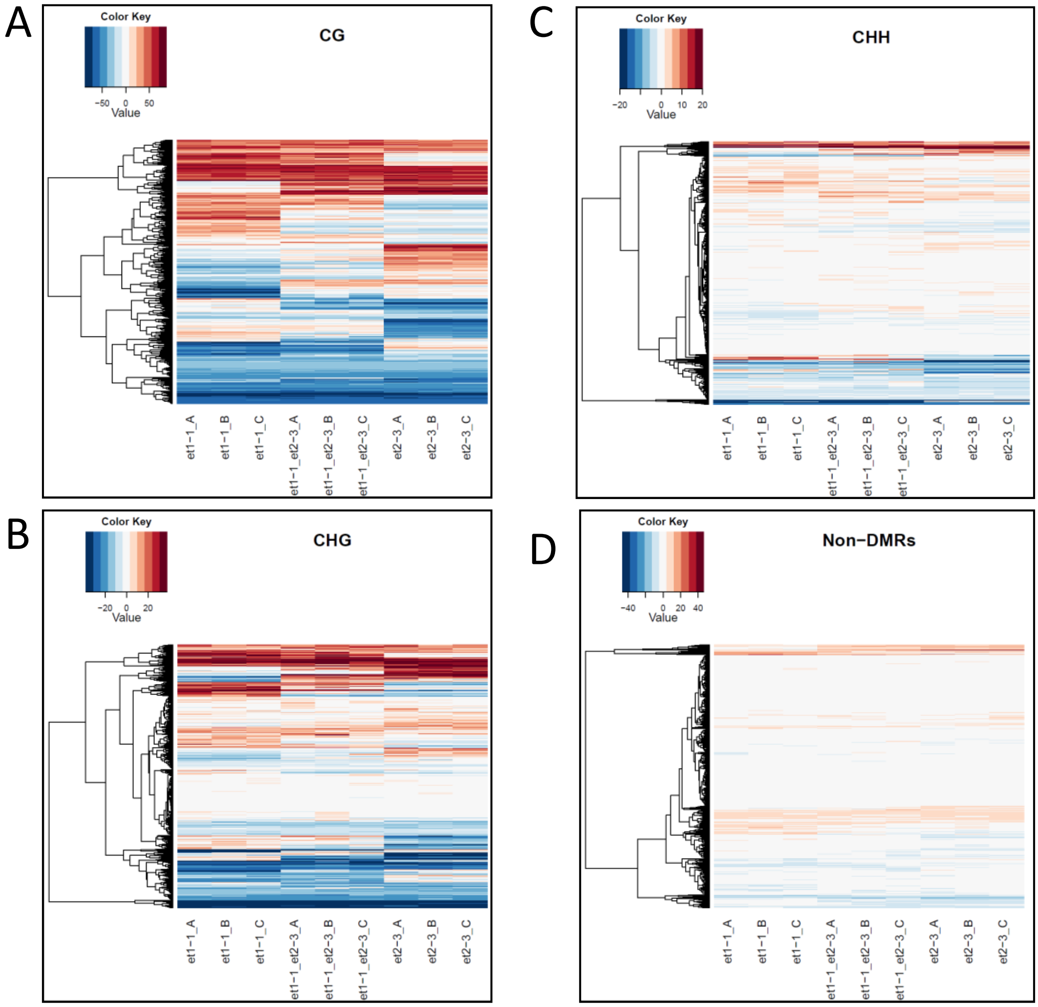

Clusteranalysis of et mutant hDMRs relative to Col-0 methylation.
Displayed is the difference in methylation rates of identified hDMRs by WGBS of *et1-1*, *et2-3* and *et1-1/et2-3* double mutant flowers.
Red color indicates increase in methylation, Blue, decrease of methylation relative to Col-0.
A difference of methylation in CG context,
B. Difference in CHG context.
C. Difference in CHH context. H = ATG.
D. Analysis of nonDMRs.
Analysis was performed in triplicate. (A, B and C)

Figure S3

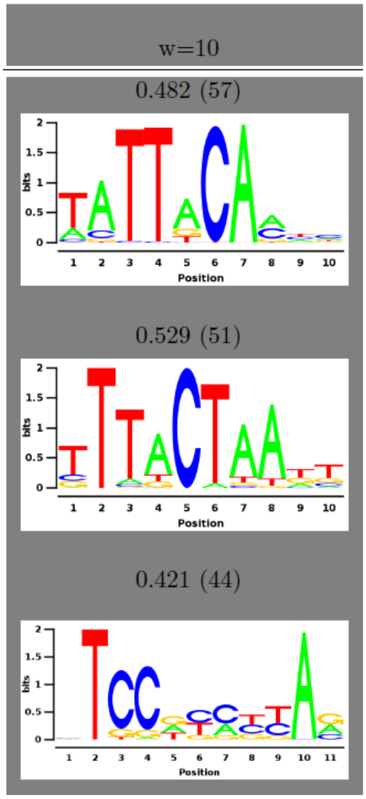

Identification of common motifs in *et2-3* highly differential methylated regions (hDMRs) (N=136) utilising the DIMONT approach.

The number of DMRs included in each motif is given in brackets. DMRs were extendet +/- 50 bps to include regions before and after DMRs. Motif length was set to w010, bgOrder=0, motifOrder=0, other parameters = default.

Figure S4

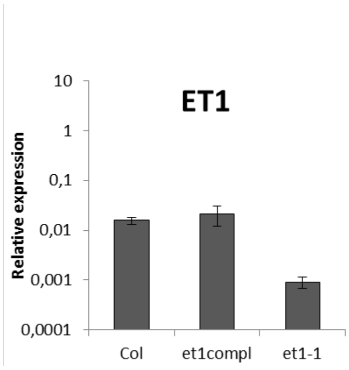

qPCR analysis of ET1 Complementation.
Displayed are realtime qRT-PCR analysis testing selecting genes for relative mRNA abundance in the Et1 / ProET1:ET1 complemented plants (et1compl). Relative mRNA abundance was compared to Col (Col-0, wildtype) and et1-1. PCRs were performed in three technical and three biologcal replicates from flower tissue. Error bars indicate the standard error.

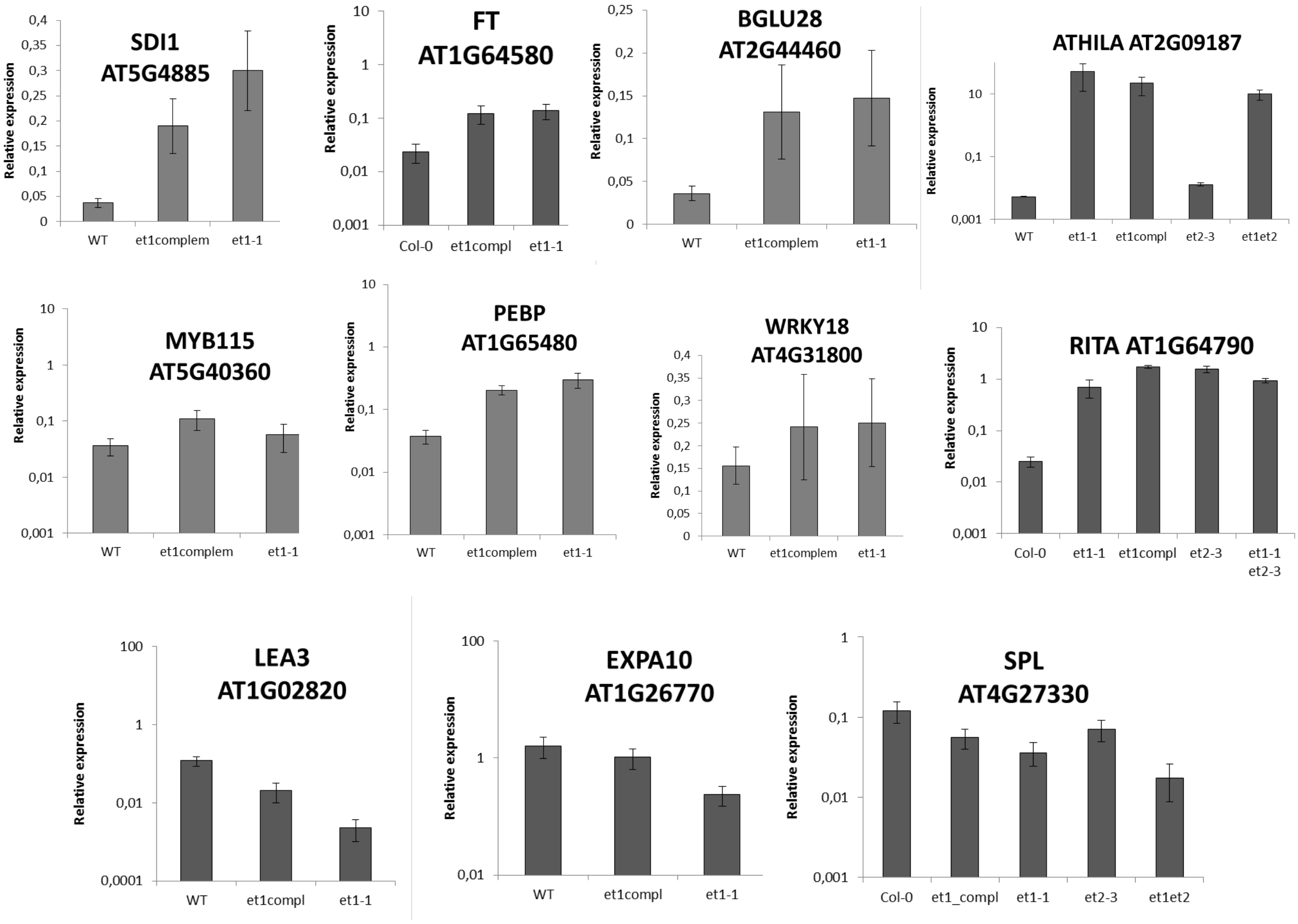

Figure S5

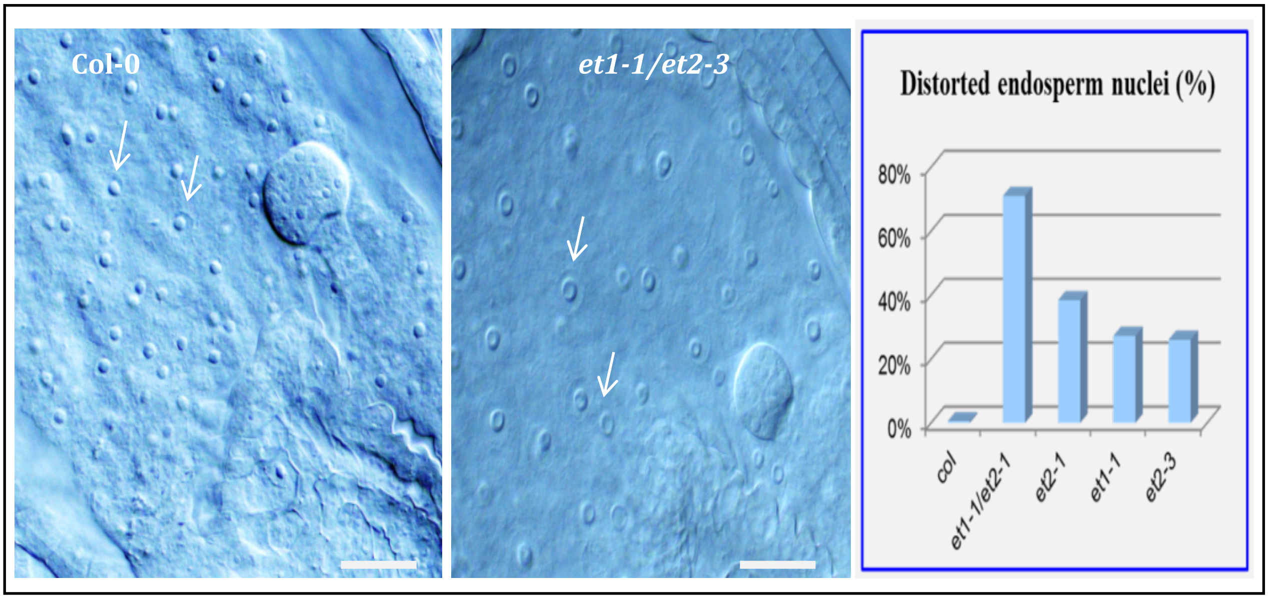

Affected endosperm differentiation in et mutants.
The nucleoli of mutant endosperm nuclei are greatly enlarged in the *et1-1 et2-1* double mutant in comparison to wild type. Scale bar: 20 µm. The morphological effect has been quantified (right panel).

Figure S6

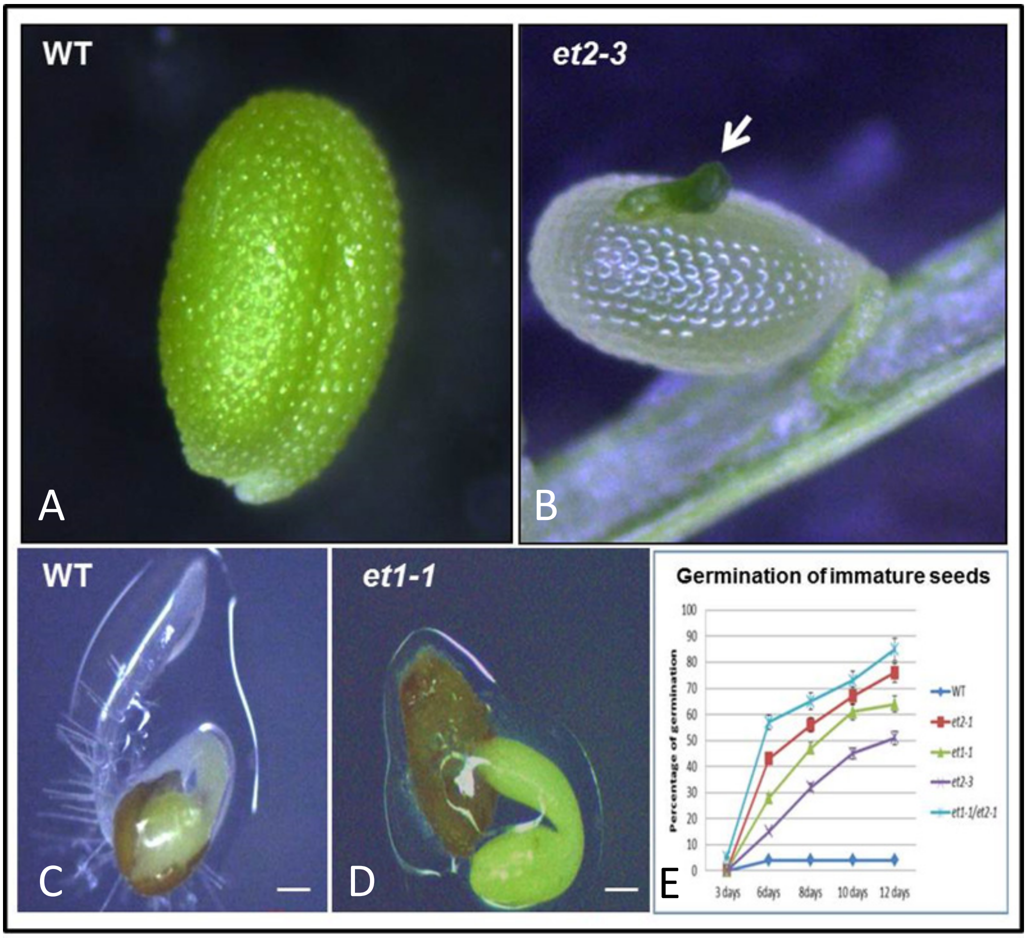

Precocious germination of et mutants.

Upper panel: (A) Wild type immature seed. (B)The immature seed of the et2-3 mutant start to germinate already within the silique. The seedling permeates the seed coat at the side of the seed with the cotyledon appearing first. Lower panel: Precocious germination of et mutants in vitro. Germinating wild type (C) and et1-1 mutant (D) seedlings: in wild type the radicle appears first, whereas the cotyledons show up first in the et1-1 mutant. (E) The phenotype has been quantified with N=200 seeds for wild type (WT, Col-0, bright blue), et1-1 (green), et2-1 (red), et2-3 (lilac) and et1-1/et2-1 (dark blue) error bars indicate +/- SD. Scale bar: 0.2 mm.

Figure S7

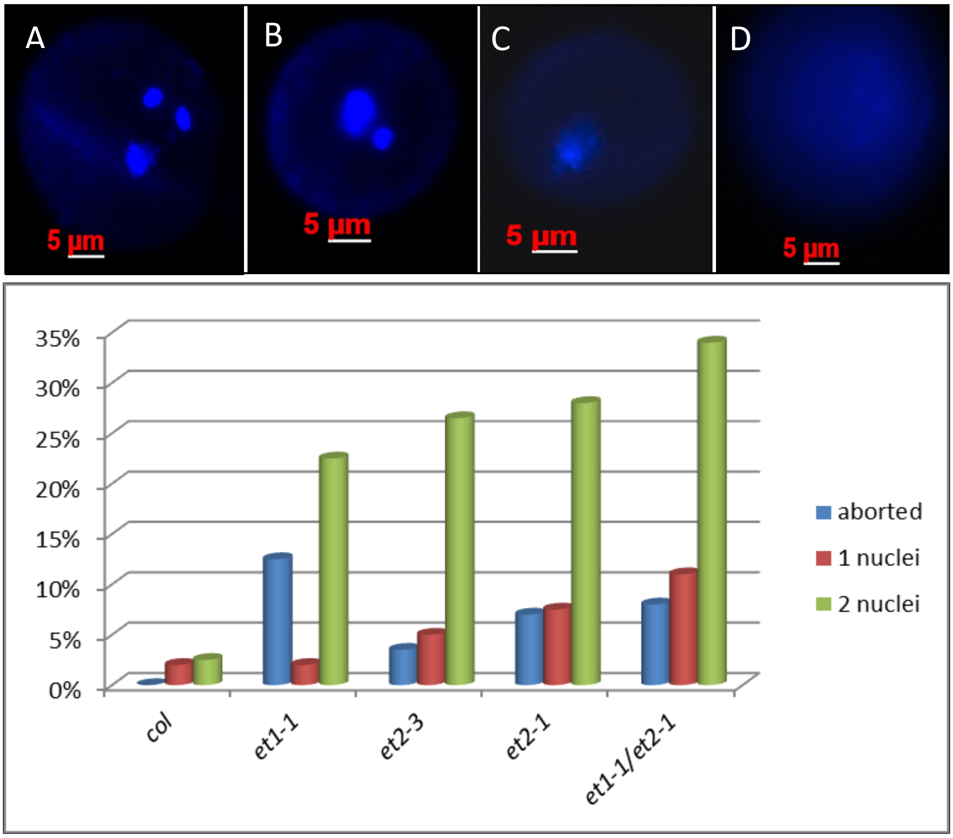

Quantification of pollen nuclei distortion.

Upper panel: DAPI stained pollen nuclei. Wild type pollen show the larger vegetative nucleus and the two generative nuclei (A). Various distortions of pollen differentiation including only one generative nucleus (B), one most likely vegetative nucleus (C) and completely collapsed pollen (D) are shown for the *et1-1* mutant.

Lower panel: Quantification of pollen nuclei distortions in *et* mutants. 200 pollen each have been analyzed.

Figure S8

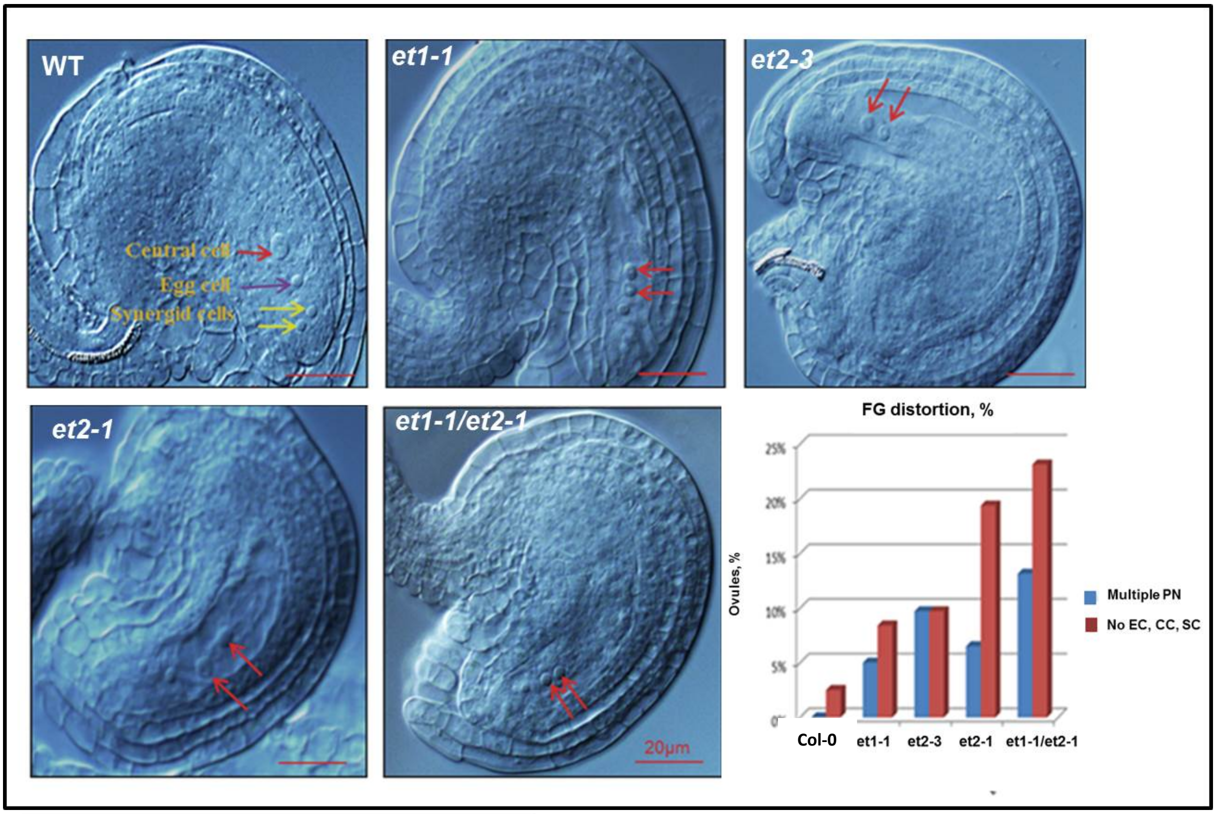

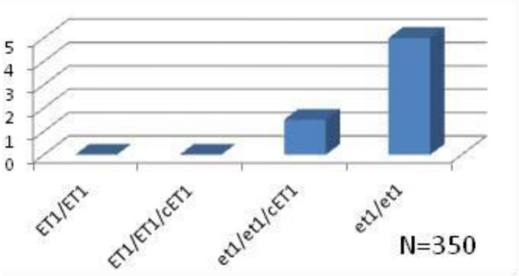

Distorted embryo sac development in et mutants.

The egg cell fuses with one of two incoming sperm cells to form the embryo, while the second sperm cell fuses with the central cell to generate the endosperm. In wild type female gametophyte, the nucleus of the central cell is homodiploid and results from the fusion of two polar nuclei. In all *et* mutants this randomly does not occur and the two polar nuclei remain non-fused (red arrows). The gametophytic distortions were quantified from Col-0 (WT), et1-1, et2-3, et2-1 and et1-1/et2-1. PN, polar cell; EC, egg cell; CC, central cell, SY; synergids, Scale bar: 20 µm.

Partial correction of the effects on non-fused polar nuclei in the gametophyte of complemented et1-1 mutants after insertion of transgenic ET1 (cET1).

Figure S9

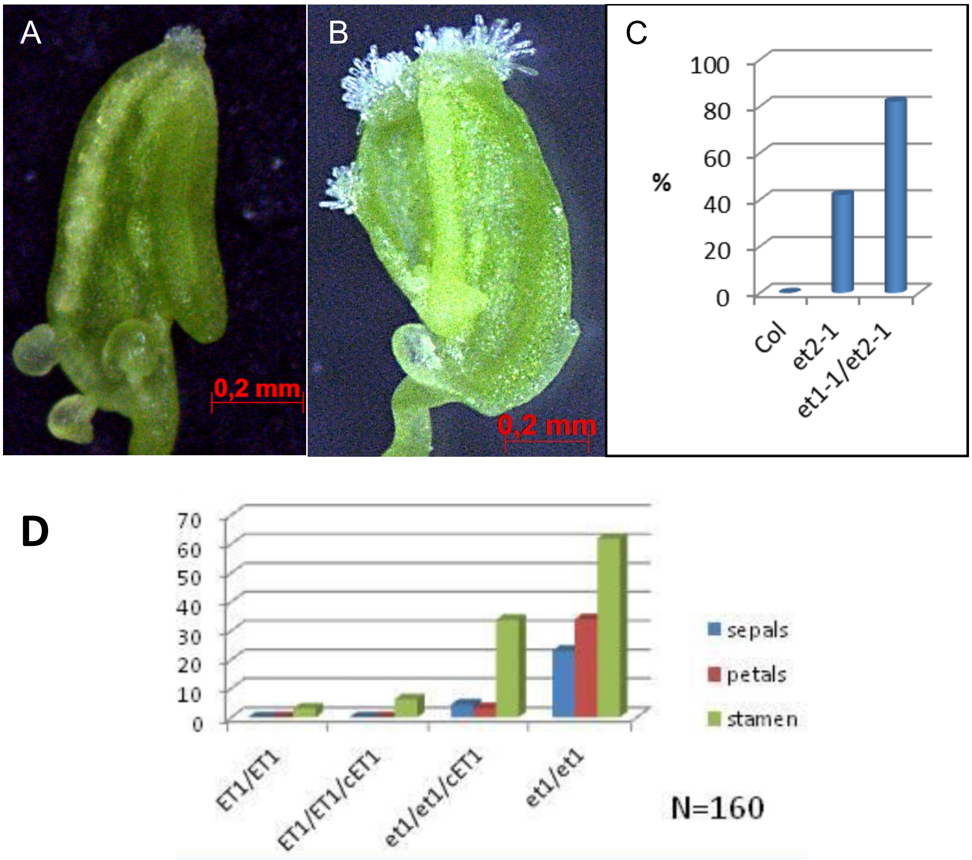

Homoeotic transformation of stamen into carpel-like structures in double mutant plants.

The homoeotic transformation of stamen into carpel-like structures in the et2-1 mutant (see above) is further enhanced in the et1-1 et2-3 double mutant with multiple ovules and stigma formation (A, B). The effect has been quantified in 180 flowers each (C). Partial phenotypic complementation of the et1-1 mutant after transformation of a 4.5 kb genomic wild type fragment. The phenotype could be partially complemented by cET1. A partial correction of effects on distorted flower organ numbers (D), partial complementation of the described effects on enlarged nucleoli in endosperm nuclei and

partial correction of the effects on non-fused polar nuclei in the gametophyte.

Figure S10

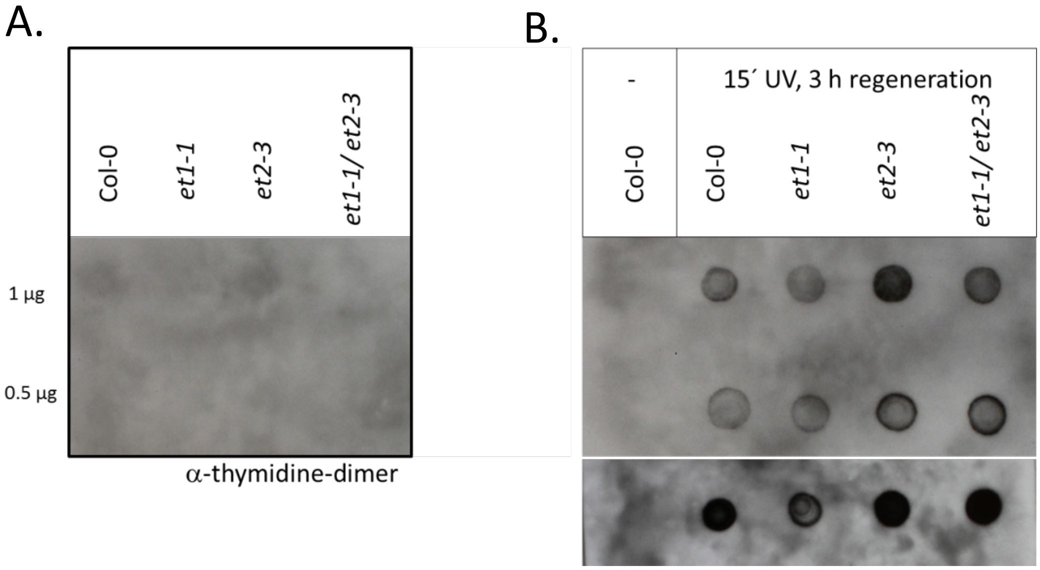

Immunodetection of CPDs on dot blotted genomic DNA extracted from leaf tissue of 2 week old plants.

A. 0.5 µg and 1 µg of extracted genomic DNA was spotted on a nylonmembran and incubated with an antibody directed against thymidine-dimers.

B. 1µg of extracted genomic DNA from plants after 15´ of UV stress and 3 h regeneration time was spotted on a nylonmembran and used for immundetection. DNA from three independent experiments were used for quantification.

Figure S11

ET1 [AT4g26170]

Organism: **Arabidopsis thaliana** / Gene: **AT4G26170** / Selected probe(set): **254017_at** / Platform: **Affymetrix Arabidopsis ATH1 Genome Array**

H3K4me3 methyltransferase

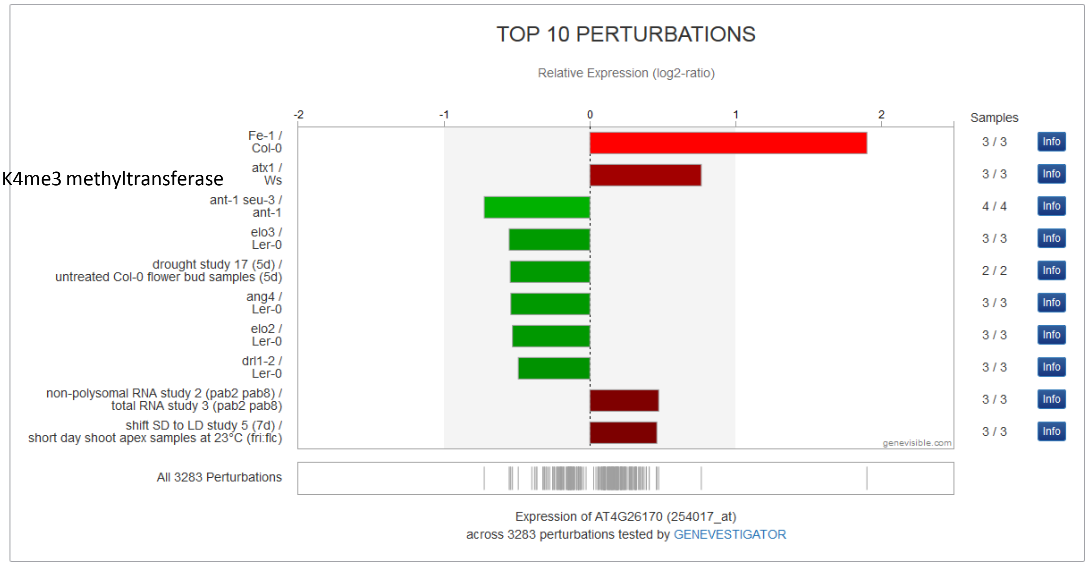

Export these results as a PNG or SVG.

Send these results to a colleague or share the *URL*.

Genevisible displays results obtained from

GENEVESTIGATOR

shaping biological discovery

ET2 [AT5g56780]

Organism: **Arabidopsis thaliana** / Gene: **AT5G56780** / Selected probe(set): **247974_at** / Platform: **Affymetrix Arabidopsis ATH1 Genome Array**

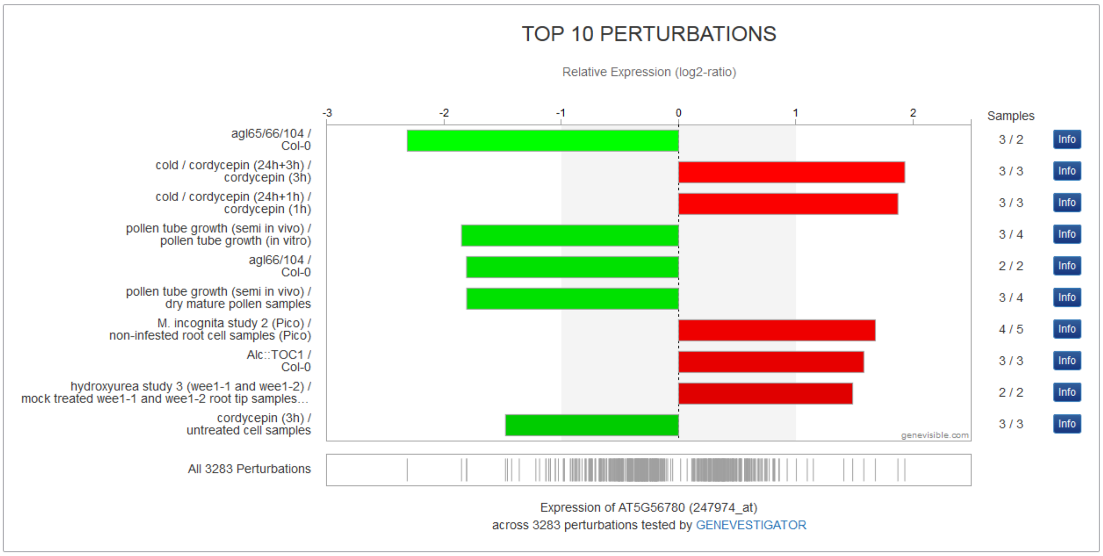

Gene expression data for ET1 and ET2 derived from public database Genevestigator.
ET1 was found to upregulated in the H3K4me3 methyltransferase *atx1* mutant relative to to Ws control and ET reacted by transcriptional activation on cordycepin and hydroxyurea treatment. Both treatments are mutagenic and cause DNA damage.
